# Improving Sustainability through Covalent Adaptable Networks in the Recycling of Polyurethane Plastics

**DOI:** 10.3390/polym15183780

**Published:** 2023-09-15

**Authors:** Edoardo Miravalle, Pierangiola Bracco, Valentina Brunella, Claudia Barolo, Marco Zanetti

**Affiliations:** 1 Department of Chemistry, NIS Interdepartmental Centre, University of Turin, Via P. Giuria 7, 10125 Turin, Italy; edoardo.miravalle@unito.it (E.M.); pierangiola.bracco@unito.it (P.B.); valentina.brunella@unito.it (V.B.); claudia.barolo@unito.it (C.B.); 2 INSTM Reference Centre, University of Turin, Via G. Quarello 15A, 10135 Turin, Italy; 3 ICxT Interdepartmental Centre, University of Turin, Via Lungo Dora Siena 100, 10153 Turin, Italy

**Keywords:** mechanical recycling, CAN, polyurethanes, polyhydroxyurethane, poly(urethane-urea), polythiourethanes, composites

## Abstract

The global plastic waste problem has created an urgent need for the development of more sustainable materials and recycling processes. Polyurethane (PU) plastics, which represent 5.5% of globally produced plastics, are particularly challenging to recycle owing to their crosslinked structure. Covalent adaptable networks (CANs) based on dynamic covalent bonds have emerged as a promising solution for recycling PU waste. CANs enable the production of thermoset polymers that can be recycled using methods that are traditionally reserved for thermoplastic polymers. Reprocessing using hot-pressing techniques, in particular, proved to be more suited for the class of polyurethanes, allowing for the efficient recycling of PU materials. This Review paper explores the potential of CANs for improving the sustainability of PU recycling processes by examining different types of PU-CANs, bond types, and fillers that can be used to optimise the recycling efficiency. The paper concludes that further research is needed to develop more cost-effective and industrial-friendly techniques for recycling PU-CANs, as they can significantly contribute to sustainable development by creating recyclable thermoset polymers.

## 1. Introduction

Plastic production began at the dawn of the nineteenth century and immediately grew exponentially because of the chemical, physical, and mechanical properties of the artefacts. The wide variety of polymers that can be synthesised has allowed the production of materials that can be used in many activities of daily life and that can hardly be replaced. In addition, relatively low production costs led to a total global production of plastics of 390.7 million tons in 2021 [1]. In contrast, it has been estimated that in 2015, approximately 6300 Mt of plastic waste was generated, approximately 9% of which was recycled, 12% was incinerated, and 79% was accumulated in landfills or the natural environment [2]. This serious issue must be addressed from a circular economy perspective, which makes recycling and reusing these materials necessary.

**Figure 1:**
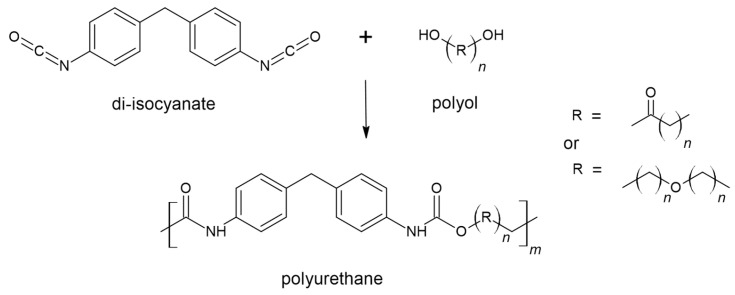
Common route for polyurethane synthesis.

Traditionally, polymers are divided into two classes: thermoplastics and thermosets. Thermosets are covalently crosslinked networks. They play substantial roles in aircrafts, vehicles, buildings, and electronics, as they are chemical-resistant and extremely durable. However, thermosets have a major disadvantage: unlike thermoplastics, they cannot be reprocessed owing to their insolubility and infusibility. Polyurethanes (PUs) can be thermoplastic and thermosetting and represent 5.5% of the globally produced plastic, placing them in sixth place for produced and used polymers worldwide [1]. The rise of PUs coincides with their discovery in 1937 by Otto Bayer et al. [3], who are rightly considered the fathers of PU chemistry and are credited with the invention of the diisocyanate polyaddition technique, which led to the production of PU via the reaction between diisocyanate and polyester diol. These two components combine to form the fundamental repetitive unit of this class of polymers, namely, the urethane group, which is produced from the reaction between alcohol (-OH) and isocyanate (-NCO), as reported in Figure 1. Still, other groups could be present in the structure, such as urea, ester, ether, and aromatic units [4]. Generally, therefore, to have a polymerisation process, diols (or polyols) and diisocyanates will be necessary [5]. Therefore, components, in appropriate proportions, will combine according to an addition reaction, which distinguishes this type of polymer [6]. Despite the potential presence of other actors in the synthesis phase, such as catalysts needed to decrease synthesis timelines [5], foaming agents, fillers, and flame-retardants, the main characteristics of the final material will be defined by alternating rigid (isocyanate) and flexible (polyol) segments [7,8]. For example, to obtain a soft elastic PU, the use of long-chain polyols will be preferred; meanwhile, rigid ones can be obtained by increasing the crosslink density by adding a trifunctional polyol. A more stretchable polymer can be obtained using long-chain low-crosslinking polyols; meanwhile, hard polymers require shorter and higher-crosslinking ones [5].

The possibility of using an exceptionally high quantity of precursors, which can also be modulated and tailored to the desired characteristics of the finished product, has made the fortune of this class of polymers. These conditions have led to the development of different types of polyurethanes (PUs), mainly attributed to the categories of rigid and flexible polyurethane foams (PUFs); thermoplastic PUs; PU ionomers; coatings, adhesives, sealants, and elastomers (CASEs); and waterborne polyurethane dispersions [5,6], which are used in various fields of industry and everyday life. Depending on the characteristics of each category, the uses can range from building materials [9], paints and surface coatings [10,11], and services in the automotive industry [12] to footwears [13], packaging materials [14], interior floor materials [15], and biomedical uses due to human biocompatibility [16,17].

Owing to its widespread commercial use, increasingly large amounts of PU waste are generated, including end-of-life waste, post-consumer waste, and processing scrap. It is estimated that the customer-produced PU waste alone ranges from 2.1 Mt to 3.6 Mt in Europe [18]. Therefore, all the processes involving the treatment of these wastes play a crucial role because, owing to the crosslinked structure of most PU products, which does not allow direct industrial recycling like thermoplastic polymers, a substantial fraction, amounting to about 50% of the total waste, is destined for landfills [7]. This disposal method creates a genuine concern as a cause of soil and groundwater pollution, originating from the PU itself or the additives used in its synthesis [19]. Some processes have been studied and implemented at the industrial level to avoid the above-mentioned related problems but, most importantly, to valorise this type of plastic waste. They are divided into five categories: mechanical, chemical, thermochemical recycling, energy recovery, and microbial degradation [19,20]. In terms of industrial importance, energy recovery and heat treatment processes rank second after landfilling [18] owing to the ease of these processes, although they are not without risk. However, the energy recovery process, mainly combustion and incineration, has some intrinsic critical issues. During combustion, harmful substances can be produced, derived from PUs themselves or from additives present in them [21]. Through thermochemical treatments, solid, liquid, and gaseous intermediates can be recovered and used for subsequent applications. Examples are pyrolysis, in which, in the absence of oxygen, PU is broken down to obtain gas and oil, and gasification, through which syngas is mainly obtained [19,20]. Instead, the objective of chemical processes is to obtain prepolymers from the polymer waste so that they can be reused in new syntheses with the possibility of obtaining materials with properties similar to the starting materials [6]. One of the most promising processes is glycolysis, which makes it possible to obtain regenerated polyols that can be used in new syntheses [22,23], which is the same objective pursued by other methods already studied, such as acidolysis [24] or, of recent interest, the application of the transcarbamoylation reaction [25].

On the other hand, mechanical recycling would be more interesting because it does not require solvents or, more generally, chemical reagents in significant quantities. Thus, mechanical recycling could be a direct reuse of PU waste. For example, the first attempts to mechanically recycle thermoset PU consisted of finely grinding the polymer and subsequently using it as filler, core component, or active part in new formulations, like adhesives [20,26]. The biodegradation of PUs, as with other types of plastics, was first observed as the environment’s response to the presence of this foreign body in the biosphere. It was observed that this could be subject to attack by organisms. Subsequently, different organisms, bacteria, and fungi, capable of showing signs of degradation, have been studied more to isolate the enzymes responsible for this action. Despite interest in the field and potential applications at the industrial level, this type of approach remains purely at the level of laboratory studies because, owing to the recalcitrance of PUs, the speed and degradation capacity of organisms remains limited and not currently exploitable [27,28].

**Figure 2:**
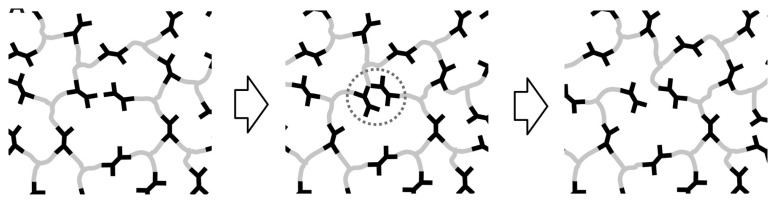
Example of covalent bond exchange process in epoxide network [30]. Reprinted from [30], and with permission from AAAS.

Despite the variety of available processes and possibility of having a starting point for new syntheses, the methods currently used at the industrial level for recycling and reusing PU are mainly characterised by downcycling rather than actual recycling or upcycling. The final products of these processes substantially do not have the same or superior characteristics as the starting artefacts. Despite increasingly stringent rules set by many jurisdictions to curb harmful industrial operations, these procedures are unappealing and inconvenient. As a result, research is essential because it can propose alternatives to present recycling and recovery procedures by examining material qualities. Specifically, as first reported in 1956 by Offenbach and Toblosky [29], PUs, when subjected to specific temperature regimes, exhibit a previously unencountered characteristic and long unexplored chemical stress relaxation behaviour, which is not typical of crosslinked materials. This type of behaviour, which had been ignored for several decades, was later proposed again in 2011 by Leibler et al. [30] for other materials, such as crosslinked epoxides, which, like PU, had traditionally proven to be unreprocessable as various types of plastics, particularly thermoplastic ones. The covalent exchange process proposed in their work is schematically shown in Figure 2, where it is possible to observe how a crosslink can be exchanged between two functional groups. In this work, it has been underlined, instead, how exploiting the stress relaxation is possible to obtain a material with characteristics similar to those of silica. Depending on the covalent bond exchange processes, this will cause a rearrangement of the network. If the mechanisms involved lean more toward associative ones, the crosslinking and, potentially, the characteristics of the material will remain constant. The behaviour of these materials, generally called covalent adaptable networks (CANs) and, in exceptional cases, vitrimers [31], was later found in other types of polymers, such as PU. This behaviour has led to the investigation of new properties related to PUs, such as self-healing, shape-memory ability, and reprocessability, which can be exploited, together with their chemical basis, for potential new processes for functionally recycling PU waste.

In this Review, we will present the current state of the art in the application of a new approach of the direct mechanical recycling of the PU family based on the exchange of covalent bonds. The literature was explored by gathering as many publications as possible containing the mechanical reprocessing capabilities of PUs. Then, because of the PU family’s breadth, it was chosen to classify several groups based on the type of basic bond present in the lattice. Based on this, it was discovered that the ultimate qualities of the artifact and reprocessing capabilities can be different. At this point, it was observed that a variety of PU materials capable of dynamicity of their own lattice have been presented throughout the years. Several chemicals have been mixed in an attempt to create more readily reworkable lattices. It was not conceivable, however, to trace back a justification for which it was possible to assert a priori that a new material was more dynamic than the old one. As a result, each product’s composition and recycling circumstances will be provided to report the combinations explored thus far and attempt to correlate the dynamism of the system with the precursors that were used.

## 2. Considerations on CANs

**Figure 3:**
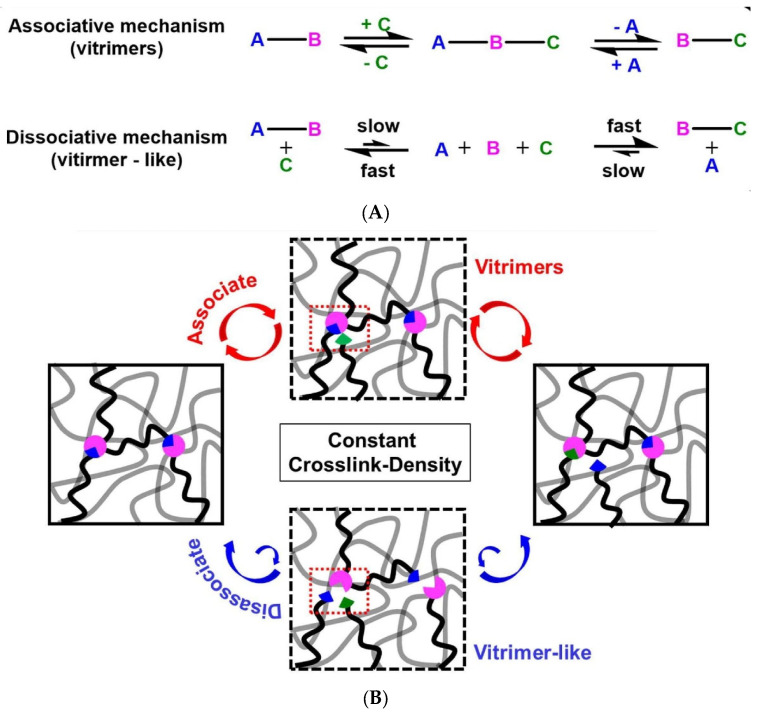

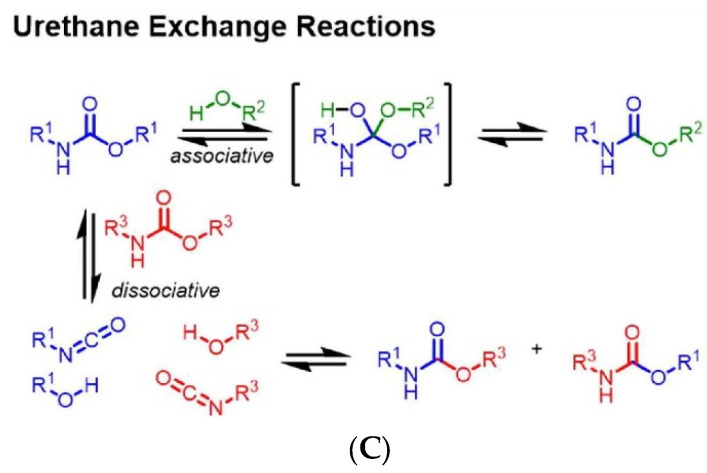
Reaction scheme (**A**) and graphical representation (**B**) of working principle of vitrimers and vitrimer-like materials; (**C**) proposed reaction for PU networks [31]. Reprinted from [31], copyright 2023, with permission form Elsevier.

Because some PU networks can be considered as materials different from the classical definition of thermosets and, thus, be counted among CANs, some considerations can be made about their related properties. Different chemical–physical and mechanical properties are closely associated with the intrinsic characteristics of this type of material, for which networks are based on dynamic covalent bonds. The dynamism of these bonds manifests itself mainly in conjunction with the application of a specific external stimulus. It can be of different types, such as the application of light, variation in pH, presence of humidity or a solvent, or appearance of redox reactions [31,32], but the most exploited at the level of recycling is heat combined with catalysis. Therefore, the presence of these bonds entirely alters the nature of the materials that were initially thermosets, making it necessary to introduce a new definition. The first attempt at cataloguing was by Lehn in the 1990s, who proposed the term “dynamers” for all the supramolecular and dynamic covalent systems, which vary their structure due to the presence of dynamic covalent bonds [33]. Then, in 2010, Bowman [34] explicitly introduced the concept of CANs and elaborated on their characteristics and properties. In particular, CANs were divided into two categories based on the dynamics of the bonds present in the network: associative and dissociative. Because two main events define the mobility of the network, the dissociation and association of the covalent bonds in the former are distinct, while in the latter, are simultaneous [33]. Their difference can be observed graphically in the reaction scheme shown in Figure 3A, while a schematic representation within a polymeric lattice can be seen in Figure 3B. A further definition was introduced by Leibler [30], who coined the term ‘vitrimers’. It refers specifically to CANs with a viscosity–temperature relationship similar to that of vitreous silica, which follows an Arrhenius-like dependence. The name was associated with this category of CANs because of its silica-like vitreous viscosity–temperature relationship in the dynamical phase of the network [30,31]. However, according to recent studies, the criterion for subdivision, the simultaneity or non-simultaneity of the cleavage and re-formation events, can vary depending on the conditions applied to the system. According to this theory, associative CANs, characterised by an almost constant crosslinking density, would have more desirable chemical and physical characteristics than those of their dissociative counterparts. However, as Dichtel [3] reported, this is not the case. Testing some dissociative CANs under the typical temperature ranges used for material reprocessing does not demonstrate different thermal properties from associative ones. This foreword clarifies that fine considerations should be made directly for individual materials under certain rework conditions rather than relying a priori on the type of bond and network one is dealing with. Since the rise in interest in this class of polymers, many dynamic bonds have been screened (e.g., esters, carbamates, carbonates, imines, acetals, thioethers, and sulphoniums [31]). Subdivisions, based on exchange chemistry, into associative (e.g., transesterification, transcarbonation, transamination, and vinylogous urea transamination [35]) and dissociative (e.g., retro Diels-Alder, urea dissociation, and transalkylation [35]) have been proposed. It remains necessary to evaluate the polymer considered in each case in order not to make wrong assignments.

A direct example is the same PUs that are the subject of this Review, which have a dynamic bond. The repetitive urethane unit presents different dynamics depending on the network or formulation in which it is inserted. It shows a dissociative mechanism when in the presence of tin-based catalysts, while in PUs with free hydroxyl groups, an associative-type mechanism is observed [31,35]. Both proposed mechanisms for this type of material can be observed in Figure 3C. However, from the point of view of reprocessability, they do not show much difference, but an associative process is thought to be more suitable for superior mechanical properties [31].

**Figure 4:**
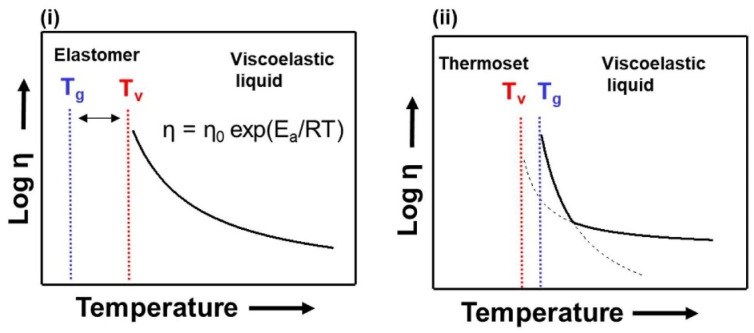
Ideal viscosity–temperature relationship of vitrimers with (**i**) T_g_ lower than T*_v_* and (**ii**) Tv lower than T_g_ [31]. Reprinted from [31], copyright 2023, with permission form Elsevier.

However, the critical point of CANs is the characteristics resulting from dynamic bonds, particularly the possibility of highlighting the appearance of new fundamental temperatures for these polymers. Namely, the upper-limit service temperature and lower recycling temperature, that is, the temperature of the topology-freezing transition (T*_v_*) [30]. In other words, above T*_v_*, the exchangeable reaction happens fast, and the vitrimer can be reprocessed and recycled; below T*_v_*, the exchangeable reaction is slow, and the vitrimer is like a traditional thermoset [30]. This temperature plays a pivotal role for heat-reprocessable CANs because exceeding this threshold sanctions the transition of the polymer from a rigid solid to a viscoelastic liquid, as rapid bonding exchanges between the chains will be possible, allowing polymer flow. This temperature is typically higher than T_g_ in the thermoset polymer case. Another important point is that the decrease in viscosity can be modelled above T*_v_* with the Arrhenius equation [32,34]. The differences in positioning between the two key temperatures for these types of materials are shown in Figure 4, along with a schematic of the viscosity trend in the system. In this way, it is possible to control, through the application of external stimuli, the exchange of bonds and, thus, the internal rearrangement of chains, which can be exploited for various purposes. For example, one of the possible applications in materials science is the creation of materials capable of self-healing and self-welding, which are essential features in building materials with lower maintenance requirements. But even more significant, is the potential to use this feature to directly rework CANs mechanically, which are initially created as thermosets, in a manner similar to that of thermoplastic materials. Mechanical recycling procedures may differ based on the network in question. The advancement of reprocessing research varies depending on the material; but, in general, they can go through welding [36], compression moulding [37], or extrusion [38]. Because these procedures are identical to those proposed for thermoplastics, their application would lower reworking costs and pave the way for the potential recycling and upcycling of these materials.

## 3. Polyurethanes

### 3.1. Thermosets

Thermoset PUs represent one of the most important classes of this type of polymer; rigid foams alone count for 38% of the total market production [7]. Their synthesis is mainly based on the combination of the two main components, polyols and diisocyanates, usually methylene diphenyl diisocyanate (MDI) and toluene diisocyanate (TDI); but depending on the types of components, ratios, and other components, e.g., chain extenders, it is possible to obtain materials with very different characteristics and usable in very different areas [7]. The feature that most people do not distinguish in this class of PUs is chemical crosslinking, which is very important. It provides the desired chemical and physical characteristics for this type of polymer but is, simultaneously, the main bottleneck that prevents easy reworking [39]. Furthermore, different types of dynamic covalent bonds have been introduced to these lattices to avoid problems in the reworking process [32]; as reported in the following studies, both PUs and methods have been developed to allow the effective reworking of this class of polymers under certain conditions.

Chen et al. [40] studied the factors that affected the property recovery of PU networks after reprocessing. They developed a PU network that fully recovers the mechanical properties of the virgin polymer. For the synthesis, tolylene 2,4-diisocyanate-terminated poly(propylene glycol) and a trifunctional triol, or tetrafunctional triol, were used in the presence of a catalyst, dibutyltin dilaurate (DBTDL), in a stoichiometric balance or excess of hydroxyl groups, to evaluate if the presence of additional hydroxyl groups could facilitate the associative rearrangement process during reworking. The reprocessing procedure was carried out first by cutting the samples into small pieces and then pressing them for 70 min at 140 °C. After homogenous films were obtained, they showed characteristics of effective network rearrangement. DMA analysis was carried out to compare the reprocessed samples with the virgin materials. It was observed that better results were obtained with the tetraol crosslinker not only in the synthesis process that obtained a polymer with a more significant E’ rubbery plateau and Young’s modulus but also in the recovery process. The values of the tensile strength, strain at break, and Young’s modulus of the reprocessed samples were close to those of the pristine samples, ensuring the total recovery of mechanical properties after the first reprocessing. Meanwhile, recoveries of 80% of the Young’s modulus and 50% of the tensile strength were achieved with the triol. In both cases, using a triol or tetraol, working with a stoichiometric excess (20%) of the polyol led to better recovery capabilities because, according to the authors, in the presence of free hydroxyl groups, the reversion of urethane was suppressed, and more chains remained in the crosslinked network at elevated temperatures. This feature also enabled excellent mechanical values after the second rework, leading to a near-total recovery.

**Figure 5:**
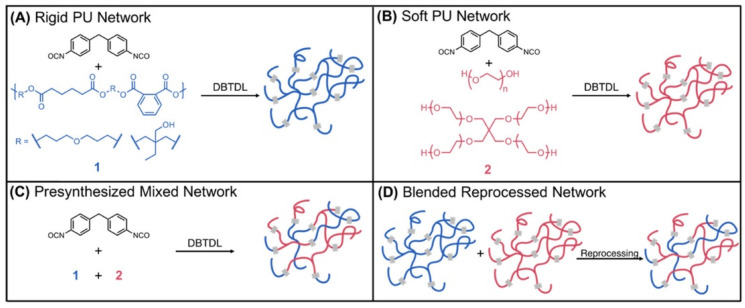
Schematic representation of the networks proposed by Dichtel et al. [41] (**A**,**B**) represent the network formed by only rigid and soft units; (**C**,**D**) underline the similarity of the combined networks obtained via the direct synthesis or reprocessing of the separated units. Reprinted with permission from [41], Copyright 2023 American Chemical Society.

Dichtel et al. [41] proposed an innovative method and compared the proposed and conventional methods for producing soft and rigid PU blends to demonstrate the effectiveness of this type of mechanical recycling. On the one hand, a classic synthesis process was carried out, creating two different PUs. The first one is tough, reacting poly[trimethylolpropane/di(propylene glycol)-alt-adipic acid/phthalic anhydride] (polyol) with 4,4-methylene bis(phenyl isocyanate) in the presence of DBTDL, tris(nonyl phenyl) phosphite and dichloromethane (DCM); meanwhile, the second one soft, reacting poly(ethylene glycol) with pentaerythritol ethoxylate under the same conditions. Also, blends of both PUs were made by varying the percentages of both polyols (25:75, 50:50, and 75:25 soft/hard polyol ratios proposed for the intermediate PUs). This synthesis process is shown in brief in Figure 5A,B, where the soft and hard PU production schemes are shown in red and blue, respectively; and, in Figure 5C, a blend resulting from using both polyols is observed. The blends have been recreated, instead, by reprocessing, in the appropriate proportions, the previously synthesised virgin PUs. Once obtained in the synthesis step, the two separate PUs are combined, as shown in Figure 5D, to form the blends, recreating the exact percentages obtained with direct synthesis. Moreover, two reprocessing processes were carried out after grinding the synthesised PUs: via two-stage compression moulding with one passage at 160 °C for 1 h and a second curing passage at 90 °C for 2 h and via extrusion at 200 °C with screws rotating at 150 rpm under N_2_ atmosphere. Surprisingly, all three intermediate blends obtained with the extrusion reprocessing method had mechanical properties that were very similar to those of the as-synthesised blends. Meanwhile, the compression-moulded one had inferior properties.

Further, the reprocessing possibility was verified by reworking the blends via extrusion by gradually adding higher percentages of PU containing the softer polyol and testing the mechanical properties. In this way, it was possible to rework different types of PU in a single step by fine-tuning the mechanical properties with the same efficiency as that of a new production, which shows potential full recyclability and reprocessability. It also emphasises that the presence of a catalyst for exchanging the carbamate bond is pivotal to allowing reworking.

In the synthesis of PUs, it is possible to use more complex reagents, which are useful for giving different properties to the final material, but they can introduce differences in the rearrangement mechanisms.

**Figure 6:**
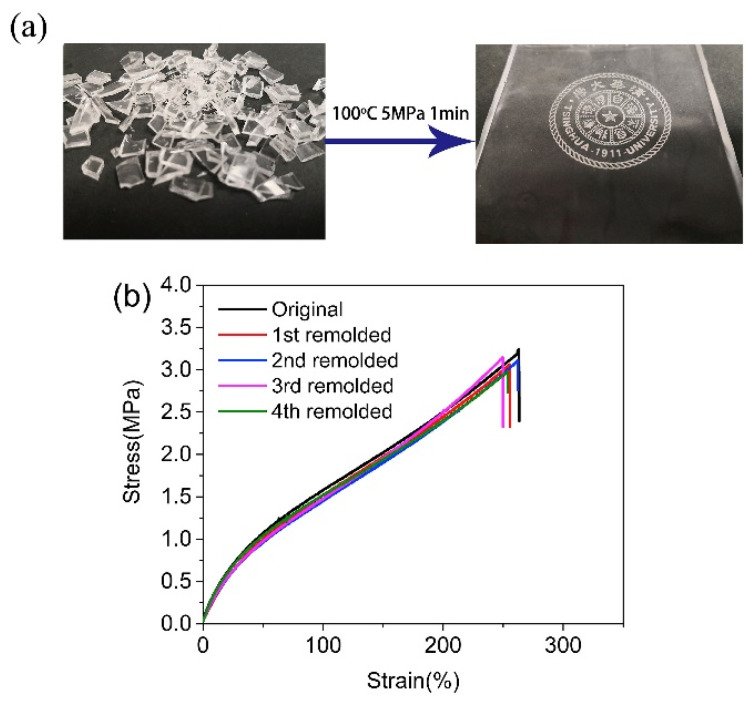
(**a**) Example of a ground sample before and after the reprocessing process via hot-pressing; (**b**) comparison of stress–strain curves of pristine and reprocessed PUs [39]. Reprinted from [39], copyright 2023, with permission form Elsevier.

For example, instead of using the urethane bond, the reversibility of the phenol-carbamate bonds has been exploited, as reported in studies by Shi et al. [39,42]. In 2019, this group [39] developed a solvent-free process to obtain a crosslinked PU that contains a particular chain extender, bisphenol S, which features a sulphone group between the two benzene rings that have a solid electron withdrawal ability that further lowers the carbamate dissociation temperature and reduces the reprocessing temperature. Meanwhile, all the other reagents are quite standard, polyethylene glycol (PEG) as a polyol, hexamethylene diisocyanate (HDI) as a diisocyanate, and HDI trimer (3HDI) as a crosslinker; an alternative catalyst, 1,4-diazabicyclo [2.2.2] octane (DABCO), is used because of its strong ability for catalysing the reaction between the hydroxyl and isocyanate and weak ability for catalysing side reactions. The reprocessing process was compression moulding, but the ground samples went through a single passage at 100 °C and 5 MPa for 1 min. Despite this, it was possible to rework the PU samples four times without observing any loss in mechanical properties compared to the virgin material, which indicates that the obtained material exploits similar rearrangement dynamics but has potential practical applications at the multiple recycling level. In Figure 6b, it is possible to observe from the stress–strain curves how, for the reworked PU, the obtained curves are almost superimposable on those of the virgin PU; and, in Figure 6a, how, even after reworking, the sample retains optical properties similar to those of the starting PU (transparency).

The same group, in 2021 [42], further investigated this type of PU, using a different kind of chain extender and varying the type of isocyanate present in the formulation to observe the variation in the mechanical properties and recyclability. In this case, bisphenol AF was used for the same purpose as that in the previous work but introduced a stronger electron-withdrawing ability to improve mobility. The isocyanates were crucial for defining the mechanical properties of the PU but negligibly affected the recycling phase. Again, compression moulding was actuated after grinding the samples for 15 min at 130 °C and 15 MPa. All the mechanical properties were close to the original ones for two consecutive cycles of grinding and compression moulding.

From these early instances, it is clear that urethane bonding lends itself poorly to dynamism and that activating components, such as electron-withdrawing crosslinkers, are required to boost its performance. As a result, one of the solutions investigated by other authors is the retention of the core PU structure to preserve the mechanical qualities of interest, while integrating secondary components that can bring dynamism to the system.

Li et al. [43] also worked with a particular crosslinker in the synthesis phase, inserting the crosslinker, 2,2′-(1,4-phenylene)-bis [4-mercaptan-1,3,2-dioxaborolane] (BDB), which is characterised by the presence of dynamic boronic ester bonds, into a network that encompasses hydroxyl-terminated polybutadiene (HTBD) and HDI. Different PU networks were made with different percentages of HTBD. Still, only the reprocessing abilities of the one containing 55% of the diol were tested, as they resulted in the most promising mechanical properties. For reprocessing, the sample was pulverised and then hot-pressed at 130 °C and 5 MPa for 1, 2, and 3 h. Although the exchange reactions are mainly based on the boronic ester group rather than the urethane group, it was possible to reprocess the material. Regardless of the reworking time, general embrittlement was observed, with decreased elongation at break and increased tensile strength compared with the original sample. According to the authors, this could be due to the macroscopic phase separation between the hard and soft regions in the pristine PU, which leads to a decay in the recovery of the tensile strength for more reworking cycles.

Zeng et al. [44] also exploited hydroxy-terminated polybutadiene (HTBD) to develop novel networks with multiple reversible interactions, such as disulphide bonds, boronic ester bonds, and hydrogen bonds. To achieve that, they reacted, in a one-pot polycondensation, HTBD and MDI with BDB and bis(4-hydroxyphenyl) disulphide (HPS) in the presence of DBTDL, obtaining different materials with different HTBD/HPS ratios. The material that showed improved mechanical properties was reprocessed via hot-pressing at 120 °C and 5 MPa for 2 h, recovering its pristine shape. After three reprocessing cycles, the material showed no variation in the FTIR spectra, a sign of the maintenance of a network similar to that of the pristine material. The material also showed good strength recovery, elongation at break, and toughness above 95%, 85%, and 70%, respectively, compared to the pristine material. The authors linked the reprocessing abilities to the incorporation of dynamic disulphide bonds and boronic ester bonds into the HTPB-based PU networks.

However, adding additional components can completely alter the system’s chemistry, shifting the focus of the recycling phase to different points in the polymer chain. For example, Zhang et al. [45] synthesised a novel PU based on an aromatic pinacol unit substituted with hydroxyethoxy groups, obtaining a material with good strength and toughness. The aromatic pinacol unit was introduced to the crosslinked poly(vinyl alcohol) (PU) during the synthesis phase and reacted with the prepolymer obtained from the reaction between HDI and polytetramethylene ether glycol (PTMEG) in the presence of a catalyst, DBTDL. Then, a trisdiol crosslinker was added to obtain the final polymer. The introduction of these pinacol units, known as radical initiators, is crucial for this reprocessable PU. The rapid formation and recombination of radicals at specific temperatures established a dynamic equilibrium that enabled the rearrangement of the crosslinked polymer chains. This rearrangement capability is shown in the reprocessing process, where it is necessary to be able to weld together chains that were not initially connected. To observe this phenomenon, the pristine polymer was finely pulverised, hot-pressed, and, subsequently, subjected to a dynamic microhardness test to measure the recovery efficiency of the recycled material. It was observed that almost complete recovery (93.6%) was achieved at 3 MPa and 80 °C for 7 h, while 119.1% recovery was detected at 100 °C for 3 h. On a small scale, it was, therefore, possible to recover the mechanical characteristics of the starting material by acting exclusively mechanically without adding additional reagents to the process.

Another example of this approach was developed by Zhang et al. [46]. They created a recyclable PU based on quinone methide secondary amine chemistry and not on the exchange of carbamate bonds. This mechanism was confirmed by observing the exchange reaction of smaller molecules and the negative control of the same synthesised PU but lacking a dynamic C-N bond related to a secondary amine. In addition, in this case, the reprocessing process was carried out via compression moulding under mild conditions, at 60 °C and 10 MPa, but for an extended period, 24 h, to obtain a defect-free reprocessed material. Finally, after three reprocessings, the observed mechanical properties were not drastically different from those of the pristine material, and T_g_ was also maintained and practically unaltered.

In PU synthesis, as in other areas of chemistry, new interests are shifting to introduce reagents and processes that respect the principles of green chemistry. From this point of view, one of the most exploited components, owing to its physical–chemical properties, which are derived from the branched distribution and multiple reactive sites in this type of molecule, is castor oil (CTO).

Yan et al. used a novel biosourced polyol crosslinker to present the first example of this type of PU [47] in the synthesis phase of PEG, CTO, HDI, DBTDL, and ethyl acetate, followed by classical PU chemistry, to observe the influence of the amount of the catalyst on the mechanical properties. The PU produced with 3 wt.% catalysts showed the best properties and was used for the reprocessing process, carried out via hot-pressing at 180 °C and 10 MPa for 2 h, obtaining a material with a smooth surface. The reprocessed sample showed good recovery capabilities, recovering 90% of the breaking strength and 85% of the elongation at break compared to the original. In addition, yield strength was observed as a result of the increase in crystallinity during the pressing process. The possibility of reprocessing PU has been associated with transcarbamylation reactions because of the absence of other dynamic bonds and through studies of polymer chains. Thus, a reprocessable material was produced based on green precursors.

Similarly, Zhang et al. [48] also developed a reprocessable PU based on CTO by adding, in the synthesis phase, a nitrogen-containing chain extender, N,N’-di-tert-butylethylenediamine (DBDA), and using isophorone diisocyanate (IPDI) and tetraethylene glycol (TEG) without using any solvent and by adding another step in the direction of green chemistry to obtain a PU with good mechanical performance. The reprocessability was assessed by pulverising the samples and then hot-pressing them at 60 °C and 10 MPa for 10 min. Materials with superior elongations at break and tensile strengths were obtained for the first three reprocessing cycles because of the more compact crosslinked structure obtained during the pressing process. The performance achieved during only the fourth reprocessing cycle was inferior to that of the pristine material. These data are good indicators of a multi-reprocessable material. In addition, the behaviour at higher temperatures was investigated, and PU turned into a viscous liquid, which could later be used for other types of recycling, e.g., injection moulding and 3D printing.

Xie et al. [49] used CTO in the synthesis step with HDI as the “hard” component and a Shiff base derived from vanillin as a multi-functional modifier in different proportions. This latter component was chosen for its hydroxyl and aldehyde groups, making it possible to be used for high-performance thermosets and vitrimer design and fabrication. The reprocessability was assessed hot-pressing the shredded samples at 20 MPa and 160 °C for 5 min. The presence of the Shiff base was crucial for obtaining a reprocessed material, as it was impossible to rework the sample with only CTO and HDI. On the other hand, for the different samples reworked up to three times, no sharp decreases in mechanical properties were observed compared to the original sample. The T_g_ and storage modulus maintained the same trend and did not change significantly.

Other innovations were made by Shi et al. [50], who introduced various types of bisphenols as chain extenders by exploiting the phenol carbamate bonds to obtain a reprocessable PU, and by Zhang et al. [51], who developed a waterborne PU by introducing synthesis formulation components like phenyl rings (as hard segments) functionalised with S-S bonds (soft segments) to obtain a PU with good performance and reprocessability. In both studies, recoveries of 100% of the tensile strength and elongation at break were obtained after a reprocessing process carried out via compression moulding, and the second study found an increase in the T_g_ of the reprocessed sample, which was caused, according to the authors, by the growth in the crosslinking density during the reprocessing process. The same synthesis technique used by Zhang et al. has been used by Liang et al. [52] and Wang et al. [53] to develop new PU thermosets. The first group reacted IPDI with 2,2-dihydroxymethyl butyric acid and PTMEG in the presence of DBTDL, adding 1,3-dihydroxyacetone and 1,4-butanediol (BDO) as chain extenders, to create a waterborne PU dispersion. Subsequently, they crosslinked the prepolymer with adipic acid hydrazide to synthesise the final material. The obtained material was cut and reprocessed by hot-pressing at 120 °C and 5 MPa for 2 min. The authors could observe almost identical stress–strain behaviour for the samples before and after three reprocessing cycles, which indicates the reversibility of the networks. Instead, the second group exploited the creation of a CO_2_-triggered emulsion as an intermediate in the synthesis. First, polypropylene glycol and IPDI reacted with BDO and N-(3-dimethylaminopropyl)-N,N-diisopropanolamine as chain extenders in the presence of DBTDL to form the prepolymer, which was subsequently activated with CO_2_ to form the final emulsion. Also, in this case, the final material was obtained by crosslinking the emulsion with 1,6-diiodohexane; the PU showing the best properties was obtained by inserting 6 wt.% of the crosslinker. The reprocessing was conducted by hot-pressing the cut samples at 170 °C for 40 min. The comprehensive properties of the material, including tensile strength, elongation at break, Young’s modulus, crosslink density, and energy dissipation ratio, were characterised. The authors observed that both the tensile strength and elongation at break of the reprocessed sample decreased, while the crosslinking density increased slightly. Meanwhile, the other properties were almost identical to those of the original sample. The authors observed a restoration of more than 80% of the properties compared to those of the pristine sample.

Other studies have used different bio-starting materials but based the reprocessability on carbamate chemistry and the same catalyst, DBTDL. Li et al. [54] used rosin as a crosslinker for a PU with a higher T_g_ than those produced classically. Hot-pressing was performed on pulverised samples at 160 °C and 5 MPa for 90 min, and the reprocessed materials showed no changes in the IR spectra, compared with that of the virgin sample, for up to three cycles. Meanwhile, the tensile strength improved (196%), and the elongation at break reduced (16%) with respect to the pristine sample. According to the authors, this feature could be related to significant phase separation between soft and hard regions during the pressing process. Kim et al. [55] proposed a mechanochemical polymerisation process, an alternative synthesis method used in green chemistry, for obtaining a PU containing 2,5-bis-(hydroxymethyl)furan (BHMF), an abundant resource derived from biomass. BHMF was reacted with other polyols, 1,6-hexanediol and meso-erythritol, in the presence of HDI and then ball milled for 1 h at 20 Hz. The resulting powder was used directly for creating PU films via hot-pressing, after which they were used to test properties and perform reworking. After being roughly cut, the obtained material was reprocessed by hot-pressing at 160 °C and 30 MPa for 4 h. Two cycles were carried out, and the storage modulus recovery was 102% for the first cycle and 90% for the second; meanwhile, the corresponding stain-at-break recoveries were 71% and 61%, respectively. This indicates that the polymer became slightly more brittle, in contrast to DMTA analyses, which showed that the crosslinking density decreased after the first reprocessing process.

Wen et al. [56], instead, tried an approach without a catalyst, using not only a bioderived polyol, tyrosol, as above, but also a bio-derived amine, dodecylamine (derived from lauric acid), to obtain a final polybenzoxazine-based PU that was fully recyclable and reprocessable. This performance was evaluated by hot-pressing the samples, previously cut into small pieces, at 130 °C and 10 MPa for 10 min to obtain an optically transparent film as the virgin sample. The same peaks were found in the IR spectra of all the samples, underlining the equality of the material structures. Also, 90% of the tensile strength and 80% of the strain at break of the film were recovered after the complete recycling. The results indicated that the catalyst-free polybenzoxazine-PU network has excellent reprocessability.

Li et al. [57] exploited the Diels-Alder (D-A) reaction to create a crosslinked PU network based on the reaction of an acetal diol derived from bioresources, 1,6-hexanediol, and a trifunctional isocyanate without a catalyst to obtain a material with superior creep resistance. The reprocessability of the material was studied at both macroscopic and molecular levels. The PU networks were cut into small pieces and hot-pressed at 190 °C and 10 MPa for 1 h. No changes were observed between the IR spectra or mechanical properties of the reprocessed and virgin material, indicating the network’s retention. At the same time, the centrality of the D-A reaction in the rearrangement process was confirmed by studying small molecules representative of the system.

**Figure 7:**
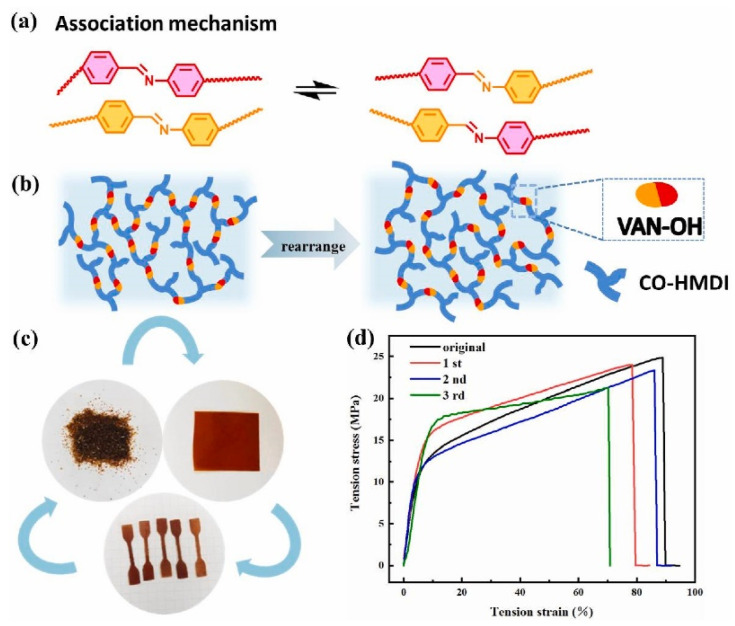
(**a**) Exchange mechanism of imine bond; (**b**) structural exchange mechanism proposed for a vanillin-bio-based PU; (**c**) example of pulverisation and reprocessing; (**d**) stress–strain curves of pristine and reprocessed vanillin-based PU [58]. Reprinted from [58], copyright 2023, with permission form Elsevier.

Sun et al. [58], instead, prepared a PU through the reaction of CTO, HDI, BDO, and a biobased vanilla diol, which contained a dynamic imine bond, as shown in Figure 7a, on which the reworkability of this material is based. The centrality of this type of dynamic bond is shown in Figure 7b; indeed, the lattice rearrangement is based on it. The samples were pulverised and then hot-pressed at 150 °C and 10 MPa for 10 min, as depicted in Figure 7c. The process was repeated three times for the PU with the best mechanical properties, and no changes were found in the IR spectra. The mechanical properties decreased slightly, as shown in Figure 7d. As observed, 86% of the tensile strength and 78% of the elongation at break were recovered for the first cycle compared with those of the pristine material. However, the possibility of obtaining a reprocessable PU network based on dynamic imine chemistry was confirmed.

**Figure 8:**
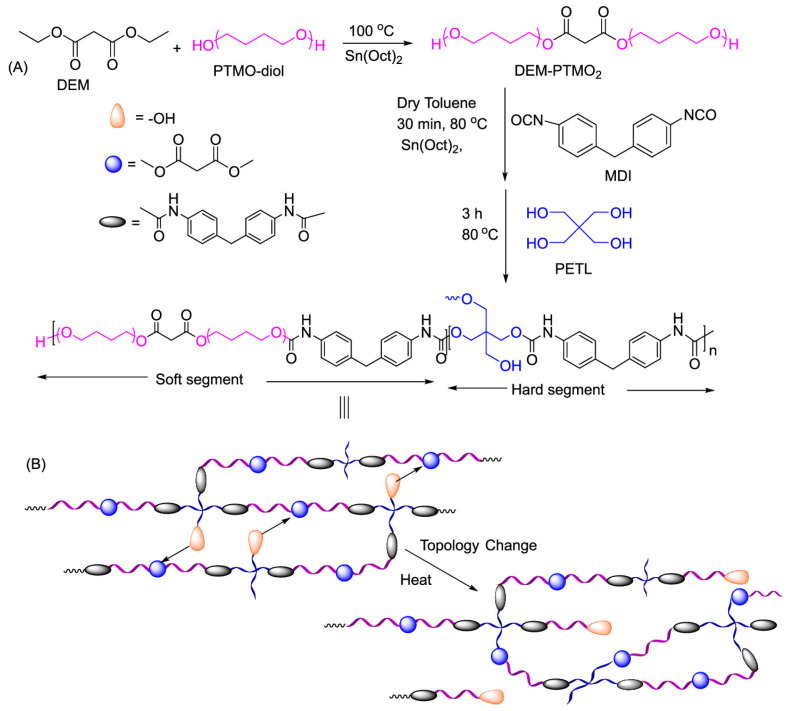
(**A**) Synthesis route of a bio-based PU; (**B**) proposed topological rearrangement upon reprocessing [59]. Reprinted with permission from [59], Copyright 2023 American Chemical Society.

Debnath et al. [59] explored the possibility of introducing dynamic ß-carbonyl carboxylate linkages in the PU formulation to render the studied materials reprocessable. The materials in question were produced starting from a low-cost biobased precursor (diethyl malonate), polytetramethylene oxides as the soft segment, and a combination of pentaerythritol and MDI as the hard segment, for which the structures and synthesis process are shown in Figure 8a. Reprocessing was carried out by hot-pressing samples, previously cut into small pieces, with a 90 kg load at 150 °C for 5 h, and recovery efficiencies of the mechanical properties were reported for the reprocessed materials in a range of 70–80% compared to the pristine sample. Despite the presence of urethane bonds, the authors confirmed that the principal mechanism on which the reprocessability of the samples is based might be attributed mainly to the ester bond exchange. Schematically, this process proposed by the authors is shown in Figure 8b.

Other methods have been proposed in addition to the hot-pressing method. An example is in a study by Zhang et al. [60], who developed a near infrared (NIR) responsive PU network based on a photothermal responsive aniline trimer suitable for 3D printing applications. In this case, the recycling was only conducted by crushing the samples and reusing them directly as a feedstock in a 3D DIW printer (Engine HR 3D printer, Hyrel 3D, Norcross, GA, USA).

### 3.2. Elastomers

Interest in the application of the recycling process to other types of PU has readily surfaced, considering the excellent results obtained for rigid PUs. For example, elastomers are a class of PU that has compositions close to those of rigid PUs, and one major difference is the crosslinking density, which is lower for this class than the rigid one. For this reason, a reprocessing process is easily achievable without major variations compared to previous cases, although it is currently less studied.

Wang et al. [61] used classic PU reagents, like poly(tetramethylene ether glycol), HDI, isophoronediamine, BDO, and HDI and DBTDL as a catalyst and added a supramolecular additive, ureidopyrimidinone (UPy), to study the effects of its introduction on recyclability and solvent resistance. The reprocessing process has been carried out via hot-pressing at 150 °C and 3 MPa for 30 min. The reprocessing ability of the samples was generally enhanced with increasing UPy content in the polymers, showing, after three reprocessing cycles, a toughness recovery of 60.9% compared to that of the original sample.

One of the areas in which elastomers play an incisive role is tire production, in which it is necessary to balance low rolling resistance, high wet-skid resistance, and high wear resistance. PUs can meet these requirements and introduce the possibility of ensuring recycling. Hu et al. [62] proposed different PUs based on different soft segments, 1,5-naphthalene diisocyanate and BDO, to meet this need. The best mechanical performances were found for the PU synthesised with polycarbonate diol as a soft segment. Recycling abilities were tested by cutting this PU sample into small pieces and moulding them three times. The tensile strength and elongation at break of the recycled samples were well maintained. Finally, the authors proposed a solution to the existing rubber tire problems and provided novel ideas for preparing next-generation high-performance materials.

As proposed by Jia et al., the ability to create flexible substrates makes elastomers suitable for creating a substrate for wearable electronic devices [63]. They proposed inserting bisphenol AF as a chain extender in a PU network created by reacting IPDI and PTMEG in the presence of DBTDL and glycerol as a crosslinker. This produced a dynamic PU elastomer that could be activated at low temperatures, based on a phenol-carbamate exchange reaction. The reprocessing process was actuated by hot-pressing the cut samples at 75 °C and 5 MPa for 30 min. Afterwards, the reprocessed samples were heated at 50 °C for 24 h to guarantee complete recombination. The IR spectra of the reprocessed samples overlapped that of the original sample, confirming that the chemical structure did not change through the process. Furthermore, the recovery of the tensile strength exceeded 95%, indicating that it was comparable to the original tensile strength. A strong electron-withdrawing trifluoromethyl group in the crosslinker proved crucial in achieving these results because it could significantly reduce the dissociation temperature of the phenol-carbamate bonds.

Dong et al. [64] synthesised a ketal-containing dynamic covalent crosslinked PU elastomer. They used poly(oxytetramethylene) glycol, MDI, a glycerol crosslinking agent, and a dihydroxyl ketal chain extender synthesised from glycerol and 2,5-hexadione. The reprocessing was conducted by pulverising the sample and then hot-pressing it at 95 °C and 12 MPa for 2 h. No changes were observed in the FTIR spectra before and after reprocessing, indicating that the reprocessing process had nearly no effect on the structure and that no side reactions occurred. An approximate recovery of 80% of the stress at strain and 65% of the elongation at break were obtained with respect to those of the pristine sample. The authors confirmed the exchange reaction based on the ketal moieties by comparing the samples to material obtained with 1,6-hexandiol instead of the dihydroxyl ketal, observing a gradual increase in the recovery efficiency with the increase in the ketal content.

Efforts to develop a biobased elastomer have been made by Xu et al. [65]. They developed a chain extender derived from the reaction between vanillin and tyrosine, both bio-derivable, and used, as a soft segment, a dimeric acid, polyester polyol, derived from oil. They used HDI as the hard segment and triethanolamine as the crosslinker to complete the synthesis condition. They obtained a crosslinked PU network based on two exchange mechanisms: imine metathesis, obtained in chain-extender synthesis, and classic blocking/deblocking of isocyanates. After being cut into small pieces, the material could be reprocessed in 30 min at 120 °C and 5 MPa. Maintenance of the main groups and the glass transition temperature and thermal properties were observed. For the mechanical properties, both the elongation at the break and tensile strength were maintained after five reprocessing cycles. The contribution of both exchange mechanisms was considered equally important because they act at two different temperature ranges. The imine metathesis takes place at around 80 °C; meanwhile, the exchange of urethane bonds at around 120 °C concurs with a very effective reprocessing performance.

This section may be divided by subheadings. It should provide a concise and precise description of the experimental results, their interpretation, as well as the experimental conclusions that can be drawn.

### 3.3. Foams

From a strictly molecular point of view, the PUs used in the production of expanded products are the same as those described so far for compact materials. Therefore, from a macromolecular point of view, the considerations made so far do not change. However, foam recycling has some peculiarities because the low density, the large surface area, and blowing agents can make the recycling process more difficult. For these reasons, a material similar to a pristine foam will not be obtained unless additional reagents are reintroduced; this leads to difficulties in comparing mechanical properties to determine the actual validity of the recovery process.

Sheppard et al. [66] emphasised this issue and tried to overcome it by extending reprocessing to post-consumer PU foams. First, they synthesised different PU materials starting from MDI and commercially available polyester polyols, eventually adding isopentane or water to obtain physically or chemically blown foams. Then, they developed a recovery method, which included soaking the material in a catalyst solution to introduce it to the network, promoting the carbamate bond exchange, and, subsequently, compression moulding or micro-compounding the dried materials via a twin extruder at 200 °C for 1 min in a nitrogen atmosphere. By comparing the mechanical properties of the obtained materials, micro-compounding led to better storage modulus and tensile strength results, which were applied to commercial foams. In the last step, the same behaviour was observed in the post-consumer foam as in the laboratory-synthesised foam, including a decrease in the T_g_ of the micro-compounded foam compared to that of the starting material.

Taking advantage of this study, Wang et al. [67] developed a PU foam bearing dynamic S-S bonds that could go through reprocessing without any passage of major concern, namely, without soaking the foam in the solution containing the catalyst. The PU foam was synthesised using polypropylene glycol, a polysulphide oligomer, which is responsible for forming dynamic S-S bonds, toluene diisocyanate, and various additives. The mixture was then recycled by compression moulding at 180 °C and 20 MPa for 30 min. Although the reprocessed material was not in the foam form, it surprisingly showed the same mechanical properties after three reprocessing cycles and the same IR spectra, which are signs of an unaltered crosslinked structure. The authors demonstrated that thermoset PU foams could be thermally processable by incorporating dynamic disulphide bonds into the backbone of the PU network.

Recently, Liu et al. [68] confirmed that inserting a sulphur-containing compound could significantly improve reprocessing. They were inserted into an industrial flexible PU foam formula based on MDI, polyether polyol, diethanolamine, and water, with 2-hydroxyethyl disulphide (HEDS) as the sulphur-containing compound. Reprocessing consisted of a “foam-to-sheet” process, including hot-pressing irregularly shaped chunks at 150 °C and 15 MPa for 30 min. The process was carried out on HEDS containing PUF and an industrial formula PUF, and only a homogenous morphology and excellent optical pellucidity were obtained in the former case. The authors linked the possibility of reprocessing the material to the presence of HEDS, as it could form a more flexible hard region combined with MDI in the synthesis phase. Disulphide and carbamide exchanges could occur in this new PU region, accelerating the stress relaxation, reducing the glass transition temperature, and improving the reprocessability. No considerations were made about the material obtained after reprocessing owing to the different forms. Still, the authors found that decent mechanical properties were achieved, obtaining a high tensile modulus (30–120 MPa) and strength (10−14 MPa), low ductility (45−100%), and a Shore A hardness in the 85−95 range, which ascribed the material to the hard rubber category.

This first section listed the materials for which the basic components enabled them to be ascribed to the class of classical PUs, divided according to how they appear in the artifact’s final mechanical properties. Although the compositions are very similar, in a few cases, there are significantly milder reworking conditions. However, the effectiveness of the rework despite a different reagent was only observed in retrospect. Nevertheless, sensitivity variations can be observed if introductions of less-canonical components are proposed. The introduction of electron-withdrawing components to the carbamate bond proved successful, allowing the creation of reworkable lattices at lower temperatures. Introducing additional dynamic bonds to the system, such as disulphide or diboron, was also effective. Both strategies can be used to design, a priori, a new potentially reprocessable PU. In the second case, however, the activation and exploitation of the urethane bond is circumvented by targeting other more easily activated dynamic bonds. In the section on foams, it was possible to reconfirm the centrality of the catalyst in the urethane bond exchange step. This, inserted in a post-synthetic manner at the rework stage, ensured that an artifact was formed. This aspect may be interesting for applying recycling methods to materials with low dynamic thresholds or, perhaps, produced to be not actually dynamic.

Furthermore, variable mechanical property recovery capacities were identified for comparable materials. Assuming that the exchange mechanisms in the various systems are similar is very interesting and would be worthy of further study, as no work has addressed this topic in depth to the best of our knowledge. Therefore, it may be useful to investigate how the composition affects the recovery capabilities to optimise the formulation at the synthesis stage to have the maximum potential recovery rate.

## 4. Polyhydroxyurethanes

Another class that has attracted interest in the production of recyclable materials is polyhydroxyurethanes (PHUs). This interest is due to historical constatations of work that observed the enhancement in stress relaxation in crosslinked PUs containing free hydroxyl groups [29,69]. Also, recent studies have confirmed the enhanced reworkability of materials obtained using a stoichiometric excess of polyols in the synthesis phase [40]. In addition, the introduction of free hydroxyl groups would significantly improve the urethane bond re-formation capabilities in the urethane exchange step for breaking and re-forming bonds, allowing the retention of the crosslink density and related properties [70].

One of the first groups to investigate this type of material was Fortman and Dichtel [71]. They observed the possibility of obtaining a reprocessable material comparable to PU without the aid of catalysts. Crosslinked PHU was prepared through the reaction of only bis(cyclic carbonate) and tris(2-aminoethyl)amine without a catalyst. Additionally, no catalyst was added during the reprocessing process, which consisted of hot-pressing at 160 °C and 4 MPa for 8 h. A 76% recovery of E’, 69% recovery of the elongation at break, and 74% recovery of the stress at break were observed compared to those of the original material. Other observations in this work suggested that PHU stress relaxation and reprocessing occur primarily through associative transcarbamoylation reactions, which could occur without a catalyst, opening possibilities for developing new and less toxic materials.

The same group also studied the properties of the final material by varying the starting precursors; in particular, they studied the thermal stability and reprocessability of PHU networks obtained from five- or six-membered cyclic carbonates without using any catalyst synthesis and reprocessing phases [72]. Six-membered cyclic carbonates were a better precursor for obtaining more stable and reprocessable materials. In addition, they observed a tensile strength recovery of around 50% compared to the original after hot-pressing at 160 °C for a reprocessing time equivalent to three times the relaxation time, which was characteristic of each synthesised polymer. Although a decrease in mechanical properties was observed from the first rework, the authors proposed that most of the reprocessed materials would be appropriate as new materials for structural applications.

**Figure 9:**
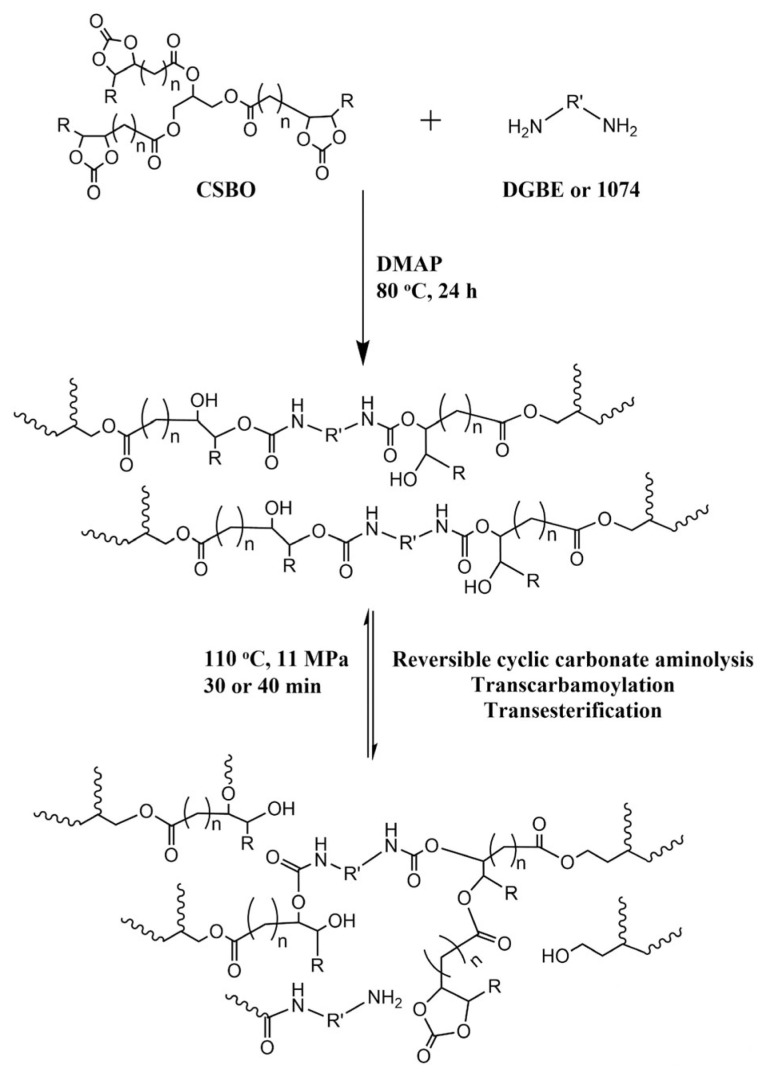
Synthesis route of a PHU and examples of exchange reactions involved in reprocessing [70]. Reprinted with permission from [70], Copyright 2023 American Chemical Society.

Hu et al. [70], instead, explored the green chemistry path by creating a PHU network by reacting carbonated soybean oil (CSBO) or sorbitol ether carbonate (SEC) with Priamine or diethylene glycol bis (3-aminopropyl) ether. Owing to the crosslinking density of the final obtained materials, only the material synthesised with CSBO was reprocessable. Its synthesis route and reworking route are shown in Figure 9. Meanwhile, the SEC-based material did not show the same capabilities due to its intrinsic structure, which leads to a highly dense network that is difficult to recover. The cut CSBO materials were hot-moulded for 30 min at 11 MPa and 110 °C, obtaining a reprocessed material with mechanical properties (Young’s modulus, tensile strength, and elongation at break) close to those of the pristine material, if not superior (for the Young’s modulus and tensile strength) for up to three reprocessing cycles. However, for all the proposed materials, a full recovery of the E’ value and crosslinking density was observed after multiple moulding steps, which was confirmed by the retention of the T_g_ value in the recovered samples.

Fortman et al. [73] also attempted an alternative method to obtain reprocessable PHU networks without the need for catalysts during synthesis and reprocessing by inserting other easily cleavable and re-formable bonds, namely S-S bonds. Next, they inserted cystamine in a formulation containing bis(cyclic carbonate monomer) and tris(2-aminoethyl)amine as a crosslinker to reach milder recycling conditions than the others reported in the literature. After grinding the obtained materials, it was possible to reprocess them up to three times at 150 °C and 5–10 MPa for 30 min. No detectable decrease in the storage modulus was observed, and a decrease in tensile strength was only observed for the first cycle (65% of the pristine), which recovered in the following processes. This approach made it possible to demonstrate the facile incorporation of disulphide-containing cystamine into PHU networks and show that these networks could be reprocessed under milder conditions.

According to the CAN theory, the presence of an excess of hydroxyl groups should enable the occurrence of simpler and more effective reworks. The excess reactive groups should enhance associative processes that maintain mechanical characteristics following reworking. Nevertheless, this could not be seen from the examples reported in the literature. Although the reworking conditions remain the same, the rates of mechanical property recovery are comparable to, if not lower than, those obtained for classical PUs. For the a priori construction of a reworkable lattice, the strategy of adding reactive groups to the lattice does not, currently, appear to be a successful one. Additionally, it does not appear that the inclusion of further labile links to the structure has significantly altered the ability of these materials to restore the properties. However, the selection of polyol or its substitute appears to be one hint for the construction of a reworkable lattice. The usage of an excessively large component appears to limit the material’s potential to be reworked. This might be considered while creating new materials.

## 5. Poly(urethane-urea)

The structure of poly(urethane-urea) (PUU) does not differ markedly from that of classical PU, as they also contain the urea bond, which differs only in the presence of a nitrogen atom instead of an oxygen atom. From a synthetic point of view, the production of these two bonds is very similar. It involves the reaction of isocyanates with hindered amines and hydroxyl groups, respectively, for the urea and urethane bonds. This leads to an accessible introduction of a new repetitive group to produce a hybrid network. However, the presence of this new bond confers the possibility for establishing new dynamic interactions in the network. They can be both weak, for hydrogen bonds that have different behaviours than those of urethane analogues, and strong, for the introduction of new reversible covalent bonds of the urea group [74]. This introduction opens the potential creation of new crosslinked polymers with different rearrangement kinetics. The possible re-crosslinkability can be provided over wider temperature ranges than those of classical PUs, due to different activation temperatures of both types of bonds. For the classic PU, the activation temperature is generally above 100 °C, as reported previously in Section 3 in this paper. Meanwhile, the dynamics and operation temperature for the urea bond are controlled mainly by the bulkiness of the substituent attached to the nitrogen, which can also lead to dynamicity at room temperature [75].

**Figure 10:**
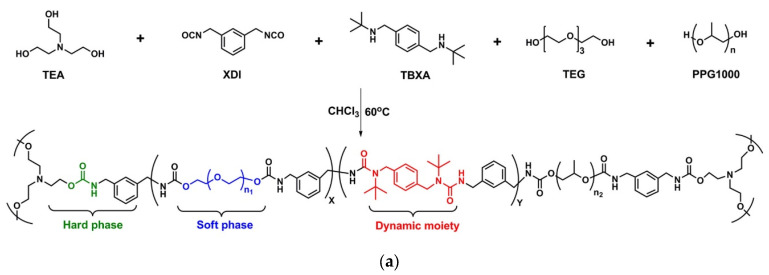

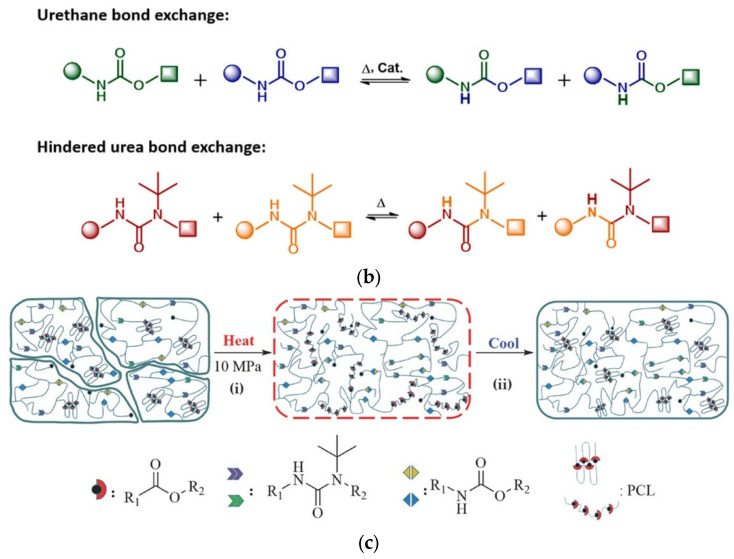
(**a**) Synthesis route of a PUU and distinction of the role of each component [80]; (**b**) main exchange reaction involved in PUU rearrangement [76]; (**c**) schematic representation of the distribution and roles of the dynamic components in network repairing [79]. Respectively reprinted with permission from [76,80]. Copyright 2023 American Chemical Society; and reprinted from [79], copyright 2023, with permission form Wiley and Sons.

One of the first groups to realise the potential of this approach was Xie et al. [76], who synthesised a PUU thermoset by reacting HDI, PEG, and glycerine (GLY) with diol, triol, and N,N-di-tert-butylethylenediamine, respectively, as hindered amines, catalysed by DBTDL. Different formulations were made by setting different ratios between the hindered urea and urethane bond (Rb) and between the PEG and GLY hydroxyl groups. Different dynamics and stress relaxation times were attributed to the networks, showing increased mobility with increasing Rb content. The reprocessability was tested only for samples with lower stress relaxation times. A schematic representation of the differences between the classical exchange of the urethane bond and the hindered urea-bond exchange is shown in Figure 10b. After being cut into small pieces, the sample was hot-pressed at 80 °C for 40 min and showed poor recovery of the mechanical properties, particularly the strain at break.

Later, better results were obtained using elastomers. In this case, Erice et al. [75] reacted poly(propylene glycol) (PPG) with IPDI and then with excess amine, 4,4′-methylenedianiline (MDA). The obtained materials were ground and hot-pressed at 150 °C and 100 bar for one hour. The recycled materials showed unchanged mechanical properties compared to those of the original material; particularly, the same stress at break was obtained. The MDA determined the recycling phase, underlining the importance of free amine groups for the efficiency of the amino/urea exchange reaction.

Inspired by spider silk, Li et al. [74] engineered and produced a supra-PU elastomer with the highest tensile strength recorded to date, using PTMEG and IPDI and an adipic dihydrazide chain extender in the presence of DBTDL. The as-obtained material could be recycled via hot-pressing at 130 °C and 5 MPa for 30 min up to five times without losing any mechanical properties, while maintaining its robustness. Zhou et al. [74], instead, worked with PTMEG, TDI, and 2-(tert-butyl amino)ethyl methacrylate, obtaining a transparent PUU network that was reworkable under milder conditions, at 100 °C and 10 MPa for 30 min, with no remarkable decrease in tensile strength and stretchability, which is a sign that this formulation can be used to fabricate sustainable materials.

Also, for this class of PUs, the possibility of turning to alternatives more adherent to green chemistry has been examined.

Wang et al. [77] developed a recyclable PUU based on the piperazine-urea bond, using a solvent and catalyst-free reaction with N-(2-hydroxyethyl) piperazine, HDMI, and CTO. The obtained material could be reprocessed up to five times via hot-pressing at 170 °C and 20 MPa for 5 min and almost entirely recovered the mechanical properties. No differences were observed in the IR spectrum and T_g_ values with respect to those of the pristine material, which is a sign of virtually no change in the network.

Also, Zhang et al. [78] used CTO as a soft segment to develop a UV-curable PUU by reacting CTO with IPDI and PEG in the presence of DBTDL. Subsequently, 2-(tert-butylamino)ethyl methacrylate, to give extra hindered urea bonds in addition to the classic urethane and urea bonds already present, and 1,3-bis(3-aminopropyl) tetramethyldisiloxane, as a further crosslinker and hydrophobicity donor, were added. The as-obtained oligomers were directly used to produce the final network, obtained via UV-curing. For reprocessing, the samples were cut into small pieces and hot-pressed at 120 °C and 10 MPa for 30 min. All the recycled samples showed higher σ, E, and ε values than the original sample. The authors linked this behaviour to incomplete material curing, which involved residual C=C groups that could lead to further crosslinking at high temperatures.

Wang et al. [79] used biodegradable poly(ε-caprolactone) (PCL) to obtain a biodegradable PUU network. They reacted PCL with N,N′-di-tert-butylethylenediamine or N,N′-diethylethylenediamine and a stoichiometric amount of (2,4,6-trioxotriazine-1,3,5(2H,4H,6H)-triyl) tris (hexamethylene) isocyanate in the presence of DBTDL to obtain a recyclable thermoset. As reported previously, the PUU approach defined by a higher crosslinking density is not as practical as that defined by lower ones. In this case, however, after a recycling process via hot-pressing at 170 °C and 10 MPa for 30 min, a good recovery of the stress at break was observed (80% compared to that of the pristine sample). Figure 10c shows the distribution of the various components and their role during the network rearrangement.

Also, Wang et al. [80] tried a catalyst-free approach for obtaining a PUU network with improved thermal stability by reacting a novel moiety N,N-di-tert-butyl-p-xylylene diamine, to endow recyclability, with m-xylylene diisocyanate and PPG. Their structure and role in the final material are shown in Figure 10a. This material can be reprocessed under milder conditions, at 80 °C and 3 MPa for 60 min, to obtain a transparent material with stress–strain curves consistent with those of the pristine material and no changes in the IR spectrum, T_g_ value, or gel content, indicating good reprocessability.

Zhang et al. [81] designed a healable and robust elastomer by introducing two dynamic bonds, disulphide linkages, and Zn^2+^/imidazole co-ordination to a thiolactone-containing PUU and subsequently transformed it into a conductive elastic sensor. They reacted polyetheramine with IPDI in the presence of DBTDL to obtain the prepolymer, then used a diol-thiolactone chain extender, and finally N-(3-aminopropyl)-imidazole and 2,2-dimethoxy-2-phenylacetophenone as a crosslinker. The obtained material showed good mechanical properties and could be reprocessed three times by cutting the sample into small pieces and hot-pressing them at 60 °C and 6 MPa for 30 min. The recovery efficiency, defined as the ratio of the breaking strength of a healed sample to that of the original sample, was 102%, 91%, and 85%, respectively, for the first, second, and third reprocessings. Also, after the insertion of carbon nanotubes to provide conductiveness, the recovery efficiency remained above 97%. The efficiency was related, by the authors, to the synergy of the dynamic bonds, through as sacrificial Zn^2+^/imidazole co-ordinations to dissipate energy and robust and reversible crosslinking disulphide bonds.

To increase the PU lattice’s reworkability, the insertion of the urea bond is suggested as an extra dynamic bond. Several authors have found it appealing to design novel materials because they can provide dynamism at low temperatures. However, something else was noticed in the creation and reworking of lattices. Compared to their PU equivalents, only few lattices show much lower recycling temperatures. One of these involves the introduction of disulphide and co-ordination bonds, shifting the dynamicity phenomenon to other components and not taking advantage of those proper to the urea-urethane bond. The introduction of bio-derived components also further complicates the system by increasing reworking temperatures. Thus, at present, the dynamism of these lattices has not yet been fully exploited, but it has the potential to allow the creation of materials with interesting mechanical properties and recyclability. Certainly, the presence of the lattice can promote the recyclability of materials, but further studies are needed to optimise its exploitation.

## 6. Polythiourethanes

**Figure 11:**
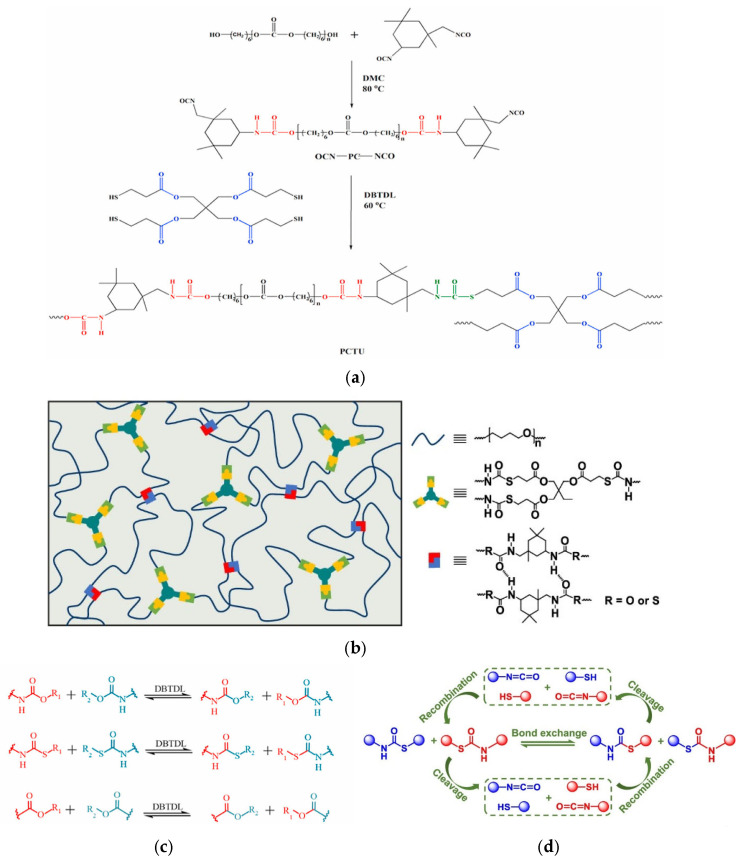
(**a**) Schematic of synthesis route for PTU networks [84]; (**b**) representation of spatial disposition of PTU components in the final network, underlining dynamic bonds and crosslinkers [89]; (**c**) exchange reactions proposed for PTU networks [84]; (**d**) proposed working paths for thiourethane bond-exchange linkers [89]. Respectively reprinted from [84,89], Copyright 2023 American Chemical Society.

Given the interest in the previously analysed class of PUs to quickly produce mechanically reworkable materials, studies have shifted to other classes of the PU family. In this regard, one of the studied classes is polythiourethanes (PTUs), which are like classical PUs but introduce a sulphur atom instead of an oxygen atom to the repetitive unit, resulting from using thiols instead of polyols. On the one hand, introducing a different heteroatom still provides similar weak interactions, e.g., hydrogen bonds; on the other hand, it provides the basis for obtaining materials with different chemical–physical–mechanical properties. For example, for the final material’s properties, the structural differences dictated by the presence of sulphur give PTUs a higher refractive index than PUs, making them more suitable for applications in optics [82]. Also, the type of exchange reaction present in thiourethanes can be compared to the one present in networks based on other bond types, as shown in Figure 11c. The processes of cleavage and recombination are very stackable to others already reported in this paper, as shown in Figure 11d. However, the characteristics that result from the chemistry in the synthesis step and the properties of the thiourethane bond itself are more important. The reaction between isocyanates and thiols that leads to the formation of this type of bond is defined as a click-type reaction, which, unlike the classical urethane reaction, is not accompanied by side reactions that can lead to the formation of unwanted products, thus complicating the system [83]. The properties of the thiourethane bond, on the other hand, are of great value in reprocessing. The thiourethane bond is characterised by lower dissociation energies than the urethane bond, which potentially makes this type of bond more dynamic and, consequently, more suitable for the creation of recyclable materials [84].

As for PUs and PTUs, different materials can be produced depending on the precursor that is used. Gamardella et al. [85], Li et al. [86], and Wen et al. [87] focused their work on developing rigid reworkable PTUs. The first group selected three diisocyanates: IPDI, HDMI, and TDI, with a rigid structure and trimethylolpropane tris(3-mercaptopropionate) (TMMP) as a trifunctional thiol to prepare PTUs with high T_g_ values. After the reprocessing was carried out by grinding the crosslinked polymers and hot-pressing them at 165 °C and 8 MPa for 2.5 h, the mechanical properties were analysed. Among all the PTUs, the aromatic and aliphatic PTUs retained 70% and 60% of the tensile strength, respectively. A slight decrease in T_g_ was observed with respect to the pristine one. This behaviour is strictly linked with the activity of the catalyst that was used (DBTDL) for the rearrangement process of the network because the PTU synthesised without it showed a significant loss in mechanical properties and a marked decrease in T_g_. The second group used the same reagents (TMMP, HDI, and DBTDL) to obtain a PTU, for which cut samples were fully recyclable up to three times via hot-pressing at 160 °C and 5 MPa for 1 h. An overall recovery efficiency above 100% was obtained for the first two times, and a recovery efficiency of 89% was obtained for the last one, suggesting that this material could be used as a component of a graphene oxide composite material, for which reworking abilities were not evaluated. The third group, instead, reacted ethoxylated-trimethylolpropane tri(3-mercaptopropionate) with HDI in the presence of a 1,5-diazabicyclo [4.3.0]non-5-ene catalyst. They reprocessed the obtained PTU by cutting it into small fragments and pressing at 10 MPa and 110 °C for 20 min three times. They obtained a material which retained 70% of the tensile strength of the original sample, demonstrating that it is possible to obtain robust networks that can be reworked under milder conditions.

Li et al. [88], instead, focused on elastomer PTUs, using a tetrafunctional thiol, pentaerythritol tetrakis(3-mercaptopropionate), and a difunctional isocyanate, tolylene-2,4-diisocyanate-terminated poly(propylene glycol) (PPG diisocyanate), and a 1,8-diazabicyclo [5.4.0]undec-7-ene catalyst. After the reprocessing at 120 °C for 20 min, the decreased mechanical properties and T_g_ were due, as proposed by the authors, to the side reactions caused by oxidation processes during this phase. Therefore, the authors decided to try minimising them by working in 10 mol% and 20 mol% excess of thiol groups. This suppressed the reversible dissociative reaction and promoted the associative exchange reaction with thiourethane groups, which rely on free thiol groups. Consequently, the E’ value, Young’s modulus, elongation at break, and tensile strength were maintained, if not slightly augmented, during three reprocessing cycles. Also, Fan et al. [89] developed a PTU elastomer by reacting an isocyanate-terminated PU, based on PTMEG and IPDI, with a trimethylolpropane tris(3-mercaptopropionate) crosslinker. The bonds and disposition in the network of the various components are shown in Figure 11b. In this case, the sample was compressed at 110 °C and 10 MPa for 15 min for the reprocessing process, showing no differences in the IR spectrum, a sign of virtually no changes in the composition of the network. Regarding the mechanical properties, no differences were found in the tensile strength of the reprocessed sample; meanwhile, an increase in the strain at break was observed. The authors link this behaviour to the slight decrease in the crosslinking density, which generated a few unrecovered thiourethane bonds. These results were compared with those for the analogous urethane network, and the recovery abilities were improved in the thiourethane polymer because of the major dynamicity of the repetitive unit.

Another method to develop PTUs is to consider their use in a specific area, such as, in this case, for recycling and upcycling carbon fibres. They can interact well with the functionalities present on the thiourethane chains to obtain a composite material that is reusable in new areas. Yue et al. [84] worked in this direction, developing a high-transparency thermoset PTU based on polyhexamethylene carbonate diol, IPDI, and pentaerythritol tetrakis (3-mercapto propionate). Its synthesis route is shown in Figure 11a as an example of PTU synthesis; also, the different components are highlighted to observe the position and role in the final network. The obtained material showed good reprocessability after hot-pressing at 180 °C and 5 MPa for 40 min, ushering in a significant part of the samples’ ability to recover the tensile strength and elongation at break above 100%. This is attributed mainly to the reduction in the crosslinking density, which increases the flexibility of the polymer. Moreover, this material showed a good affinity with carbon fibre to obtain reinforced materials for automotive fields, ensuring a reasonable recovery rate for possible future reuses.

Cui et al. [90] developed a PTU network for carbon fibre and glass adhesive purposes. They started by reacting different isocyanates m-tetramethylxylylene diisocyanate, IPDI, and HDI with trimethylolpropane tris(3-mercaptopropionate) and N, N-diisopropylethylamine to obtain optically transparent PTUs, which were reprocessed via pulverisation and subsequent hot-pressing at 3 MPa and 120 °C for 1 h or at 3 MPa and 80 °C for 30 min. For all the samples, the same Young’s modulus was observed in the reprocessed sample as in the original, and up to 80% of the tensile strength of the pristine sample was retained. These formulations were combined as an adhesive with carbon fibres but, most interestingly, with glass, creating a PTU-coated glass with properties similar to those borosilicate glass.

Alternatively, Erice et al. [91] exploited the dynamism of S-aromatic thiourethane crosslinks and catalyst activation to produce a novel injectable and recyclable PTU. They used polypropylene glycol and IPDI to obtain the prepolymer. They then reacted it with two different thiol hardeners, 1,4-benzenedithiol and glycol di(3-mercaptopropionate), but only the PTU synthesised with the first hardener in the presence of DBTDL showed reprocessability. The reprocessing process was carried out by hot-pressing the finely ground samples at 160 °C and 100 bar for 1 h. The recovered material showed a higher tensile strength than the pristine one due to different packaging applied during curing. Finally, the authors prepared a possible alternative for PTUs that is directly processable by injection moulding and completely recyclable.

It was hypothesised that using thiols rather than polyols in PTUs might result in a more effective and energy-saving dynamism. The research mentioned above shows that temperatures and timing do not appear to be significantly lower, nonetheless. The lability of the thiourethane bond should, in theory, result in a noticeably reduced level of recycling conditions, but this is not observable. Additionally, different reworking temperatures and timings were used on some materials with the same composition, producing diverse outcomes. As a result, it is obvious that dynamics exist that have not yet been discovered or investigated, as an equal composition would imply equal recycling conditions. The utilisation of surplus thiol units to prefer associative processes over dissociative ones for the synthesis of more readily reprocessable materials appears appealing for PTUs. Additionally, given that the usage of injection moulding has been reported by certain authors. Further research should be conducted to determine whether this is an isolated incident or whether similar or unrelated materials can also benefit from this technique.

## 7. Composites

Because Pus are also widely used as a matrix of composite materials, the possibility of exploiting the reprocessability of CANs has also been studied for this class of materials.

Zhao et al. [92] studied the reinforcement of PU composites by the insertion of polyethylene nanocrystals into the network in different ratios. The composites were synthesised in two steps, creating dihydroxy-terminated PU and PE separately and mixing them in the correct ratio. The first is prepared from MDI, PTMEG, and BDO in the presence of DBTDL. Meanwhile, the second, produced by reacting 2-butene-1,4-diol with cyclooctene via the ROMP reaction, was adequately reduced. The ultimate reaction to obtain the composite was carried out in the presence of DBTDL and zinc trifluoromethanesulphonate. The reprocessing method consisted of cutting into small pieces the obtained samples and hot-pressing them at 70 °C. It was then observed that the mechanical properties were unchanged even after three recycling processes at different PU/PE ratios (e.g., 100, 90/10, and 80/20). The importance of including PE nanocrystals can be observed in the reinforced PU lattice, improved mechanical properties, and recycling. The authors proposed that the presence of PE in the lattice caused a decrease in E’ and G’ and an increase in viscosity, promoting the reworking of the material.

Yang et al. [93] introduced furfuryl-modified silica particles in a high functional-group grafting ratio, produced by the sol-gel process, to a maleimide-terminated PU elastomer to obtain a thermoreversible organic–inorganic network based on the D-A reaction between the silica particles and PU network. The silica particles and PU network precursor were produced separately and only mixed in a second moment, using the silica particles as a crosslinker between the PU chains. The obtained product underwent two types of recycling, one by solution casting and the second via hot-pressing, at 10 Mpa and 130 °C for 10 min. In both cases, the recovery of the tensile strength was greater than 75% of that of the original material, indicating the versatility of recycling this material and the possibility of producing reprocessable hybrid materials.

**Figure 12:**
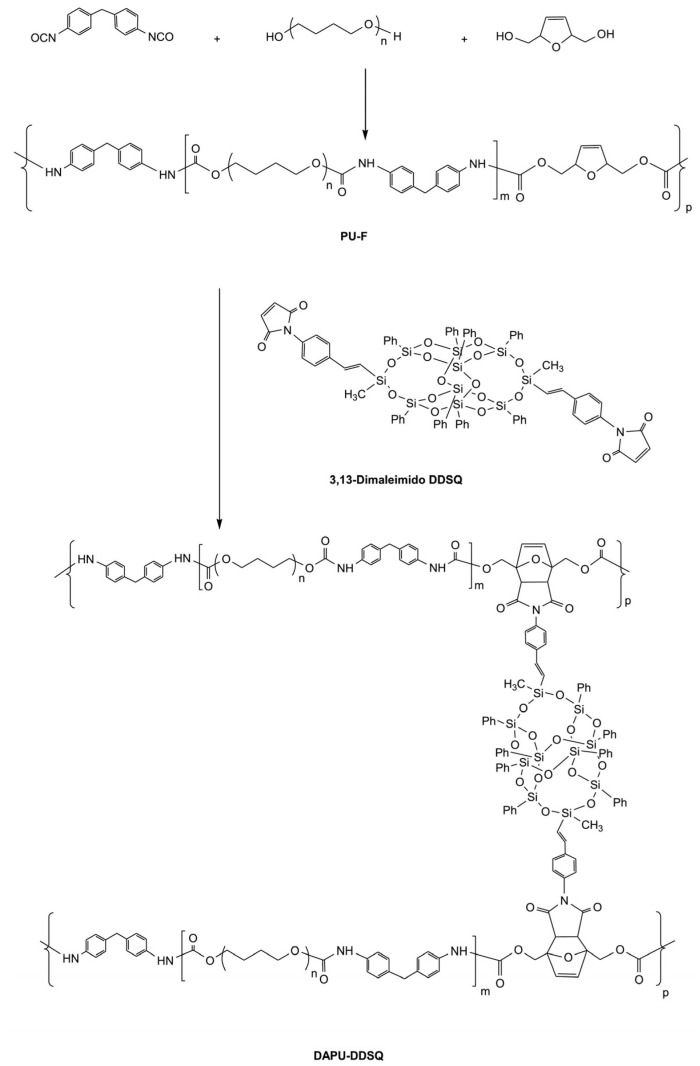
Synthesis route of a composite PU and example of using an external agent (organo-silicon) as a crosslinker [94]. Reprinted from [94], copyright 2023, with permission form Elsevier.

Given the demonstrated effectiveness in the application of D-A for obtaining reprocessable networks, other work has relied on the same reaction, such a study by as Zhao et al. [94]. They used a difunctional double-decker silsesquioxane (DDSQ) to crosslink a PU synthesised from MDI, PTMEG, and 2,5-furandimethanol (as a chain extender). DDSQ is vastly used in producing organic–inorganic hybrids as nano reinforcements to improve the properties of organic polymers; an example of its introduction at the synthesis stage is shown in Figure 12. Reprocessing properties were tested by hot-pressing small pieces of the virgin material. The authors observed that the stress–strain curves of all the obtained specimens were almost superimposable on those of the starting materials, regardless of the different proportions of the reagents used to prepare them. Finally, the authors proposed the centrality of the D-A reaction in both the dismantling of the network at elevated temperatures (130 °C) and in the regeneration phase at lower temperatures (<90 °C), as the main-chain furan rings of the PU reconnected with the maleimide groups of the DDSQ.

Other types of inorganic fillers and reinforcers are used for developing new polymer networks, such as Fe_3_O_4_ nanoparticles (NPs), as studied by Li et al. [95] for the creation of a nano-reinforced PHU network. They previously functionalised Fe_3_O_4_ NPs by grafting poly (2-oxo-1,3-dioxolane-4-yl)methyl methacrylate to facilitate dispersion in the PHU lattice and react a five-membered bicyclic carbonate monomer [4,4′-bi (1,3-dioxolane)]-2,2′-dione with a trifunctional polyether amine. The obtained composite was reprocessed by hot-pressing at 150 °C for 3 h to produce a consolidated recycled specimen. To compare the efficiency of the reprocessing process, the ratio was observed between the integral area of the strain–stress curves for the reprocessed and pristine samples. After one reprocessing cycle, this parameter dropped from 98.2% for the plain sample to 81.6% for the sample containing 30% NPs. Therefore, despite the presence of NPs, the network was reprocessable as well, even if only slightly. The authors proposed that the incorporation of Fe_3_O_4_ NPs influenced the decreased recovery of mechanical properties, which decreased the fraction of reversible covalent bonds in networks and restricted the segmental motion of PHUs; therefore, the activation energy of the dynamic exchange reaction increased.

Another group that used the D-A reaction to obtain a rearrangeable composite PU network is Du et al. [96]. They used this reaction to unite functionalised reduced graphene and a prepolymer made of PTMG, polyhydroxyalkanoates, N-(2,3-dihydroxypropyl)-maleimide, as a chain extender, and the crosslinking reagent of furan-terminated hexamethylene diisocyanate trimer. Graphene was inserted in this composite because it could absorb and convert near-infrared light to thermal energy and improve the temperature of polymer matrix materials. It could enable the occurrence of D-A reactions without the need to apply heat, which was a novel point of this work. The reprocessing of this composite involves a first phase of comminution and positioning in a press at 20 MPa, but unlike the work reported previously, the whole sample is subjected to NIR irradiation for 10 min. This process was compared to solution-casting reprocessing, and the results for the recovery of the tensile strength were inferior but still excellent, recovering up to 82% of the original sample. This work may have promising applications in light-responsive thermoset shape-memory materials.

Also, bioderived reagents have been exploited. Qi et al. [97] controlled the potential of nanosized lignin as a polyol crosslinker in the synthesis phase and for the final properties of the PU, ensuring good thermal, mechanical, and mechanical reprocessability properties. The PU network, also synthesised with PEG, HDI, and DBTDL, was reprocessed by cutting the samples into small pieces and then hot-processing them at 120 °C for 1 min. For the reprocessed materials, the tensile strength, Young’s modulus, and elongation at break, respectively, were 82.6%, 82%, and 53% compared to those of the pristine material, are a sign of the occurrence of transcarbamylation reactions catalysed by DBTDL, which led to the rearrangement of the lattice, ensuring a material performance typical of that of vitrimers. Moreover, a reduction in T_g_ of 5 degrees (−16 °C pristine; −21 °C reprocessed) was noticed owing to the decrease in the dynamic crosslinking network, which is the main cause of the lowering of mechanical properties. Recently, Huang et al. [98] also used the same reagents, polytetramethylene ether glycol, PEG, HDI, and nanosized lignin to obtain a reprocessable PU network with anti-aging properties. The reprocessing was carried out by cutting the material into pieces and hot-pressing them at 165 °C and 10 MPa for 20 min for three times, maintaining 85% of the strain at break and 63% of the stress at break in the reprocessed material with respect the pristine one.

Li et al. [99] also exploited the polyhydroxyl structure of lignin as a raw material with IPDI, polytetramethylene ether glycol, and DBTDL, obtaining films with excellent transparency, UV resistance, high elastic recovery and good mechanical properties. They produced samples with different percentages of lignin, but they only used samples containing 0% and 95% lignin for reprocessing. They reprocessed the materials 10 times, and the mechanical properties were almost fully recovered for the sample without lignin. Meanwhile, for the lignin-containing sample, the opposite trend was shown. In the authors’ opinion, that was due to the low efficiency of the urethane bond transesterification reaction caused by the lignin’s hindrance. The increase in the lignin content in the formulation had a visible impact on the mechanical properties, leading to more brittle materials and lower reprocessability. Lignin could be a good starting point for developing PU materials containing fewer polyether polyols and more sustainable counterparts with interesting characteristics. However, its role in the reprocessing process could prove to be a disadvantage.

More recently, Wang et al. [100] similarly used lignin, taking advantage of its rich polar functional groups and phenolic hydroxyl groups, and inserted Zn^2+^ cations during the synthesis to create reworkable PU elastomers with a reversible dual-crosslinking network. The dual-crosslinking network consisted of dynamic noncovalent bonds (due to the presence of the cation) and dynamic phenol−carbamate bonds. PTMEG reacted with HDI in the presence of DBTDL. In the following steps, 3,5-diamino-1,2,4-triazole was added as a chain extender, obtaining the prepolymer and lignin as a crosslinker to obtain the final elastomer. Reprocessing was conducted by pressing small pieces of the pristine network at 160 °C. The authors observed the possibility of repeating, up to four times, the recycling process while maintaining up to 85.4% of the tensile strength and 95% of the elongation at break with respect to the pristine sample. In this process, the phenol-carbamate exchange was primarily exploited; meanwhile, the second reversible noncovalent bonds were observed to be more important in the NIR-stimulated self-healing ability, which could also be applied to the former in future applications.

Zhao et al. [101] exploited lignin to develop an innovative PHU network that can be synthesised via an isocyanate-free, solvent-free, and catalyst-free mechanism. They reacted a six-membered bicyclocarbonate with diamines (Priamine 1074) and lignin as a crosslinker, obtaining a functionalised hydroxyl PU matrix characterised by a dynamic covalent network. The obtained material could be reprocessed by hot-pressing at 160 °C, maintaining a tensile strength, elongation at break, Young’s modulus, and tensile toughness, respectively, of 83.1%, 88.5%, 89.5%, and 71.9% compare to those of the pristine sample, despite the 30% lignin content in the analysed sample. The authors mainly linked the decrease in mechanical properties to heat-induced side reactions in reprocessing.

Following the introduction of biopolymers, as reinforcing agents, to PU networks, Ge et al. [102] used nanocrystalline cellulose (NCC) to obtain a reinforced PHU. A previously surface-grafted NCC with N-vinylpyrrolidone (NCC-PVPy) was used to crosslink a PHU obtained from a novel novolac epoxide-based cyclic carbonate reacted with trifunctional polyetheramine. The PHU network was reprocessed by hot-pressing at 160 °C for 4 h. For example, in the nanocomposite containing 40 wt.% NCC-PVPy, there was up to 60% retention in the fracture energy (a parameter proposed by the authors to indicate the remaining mechanical strength after reprocessing) after the specimen was reprocessed twice. As a general trend, the loss in mechanical properties increased with increasing content of NCC-PVPy. According to the authors, this was due mainly to the modifier itself, which did not contain the motif responsible for the reprocessing properties, and to its fine dispersion, which would exert a significant reinforcement effect on PHU matrices but could decelerate the reaction kinetics of dynamic covalent bonds at elevated temperatures.

It can be advantageous for numerous reasons to develop composite materials that use rearrangement dynamics for reworking. For instance, it is possible to obtain materials with unique characteristics from those of their PU equivalents. Additionally, it is possible to incorporate elements that respond to various stimuli, enabling reworking using various techniques, such as UV light irradiation. This approach might be effective for cutting delays and potentially even related expenses. It becomes clear, nevertheless, that the use of large biobased fillers, such lignin, can cause more issues during the reworking process than anything else. As a result, it is essential to investigate this matter more deeply and carefully balance the introduction of various components to benefit from the advantages they provide.

## 8. Others

Further investigations have been carried out on other types of networks not ascribable to the categories previously reported. The aim is always to obtain recyclable materials with special chemical–physical and mechanical characteristics, potentially usable in industrial fields or to find alternatives to materials already proposed but improved in some aspect (e.g., ease of reworking).

Gao et al. [103] took inspiration from PTU and PUU and developed self-healable polysulphide-based PUs starting from a polysulphide oligomer as the soft segment, IPDI as the hard segment, and 2,5-diamino-3,6-dimethyl-mercapto as the chain extender. According to the authors, the idea behind the choice of these precursors was to achieve a material with better mechanical properties than polysulphide-based polymers by exploiting the known potential of PU networks. The obtained material showed a tensile strength three times higher than the benchmark considered by the authors. Additionally, it was maintained without significant losses after three recycling cycles, carried out via hot-pressing at 100 °C and 5 MPa for 30 min. These results are evidence of the potential to obtain a recyclable sulphur-based polymer that is not sensitive to thermal oxidation processes or exposure to air and, therefore, is applicable from a sustainable industry perspective.

**Figure 13:**
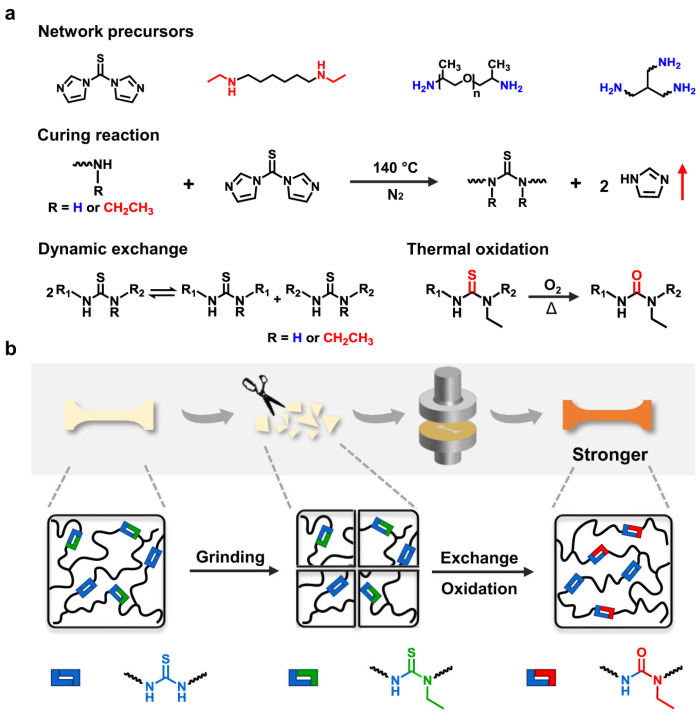
(**a**) Precursors used in synthesis and proposed reaction involved in the formation of the PU network; (**b**) representation of the reprocessing process and oxidation-strengthening process of the network [104]. Reprinted from [104], copyright 2023, with permission form Springer Nature.

Along the same line, Feng et al. [104] developed a thiourea-based PU to take advantage of the intrinsic characteristics of this bond and obtain a chemical hardening phenomenon during the reworking phase. The network was prepared by reacting poly (propylene glycol) bis (2-aminopropyl ether), N,N-diethyl-1,6-diamino hexane, trimethylolpropane tris (poly (propylene glycol), amine terminated) ether, and thiocarbonyldiimidazole. For reprocessing, the polymer was ground into small pieces, hot-pressed (4 MPa, 30 min, and 140 °C) and then conditioned at 140 °C in open air to obtain all the benefits of the oxidation conditioning. In this type of PU, the repetitive unit is sensitive to oxidative phenomena. Internal changes to the chains could occur, such as the loss of sulphur and oxygen insertion, which lead a material with different mechanical properties. The sulphur atom is replaced by oxygen atoms during the reprocessing phase, transforming the thiourea group into a classic urea group with other strong bonds (involved in dynamic exchange) and weak interactions (H-bonds). As schematically shown in Figure 13a,b, the exchange process is based on the thiourea group to exploit the sulphur chemistry. In the second phase, thermal oxidation occurs, which introduces different groups to the network and changes its properties. A greater Young’s modulus was obtained for major conditioning time scales, and these values, along with those of the tensile strength, increased with increasing number of recycling cycles (confirmed for up to three cycles). This approach can be a winning strategy for producing new recyclable materials so that real upcycling can occur.

The route other authors have followed is based on an additional, less-studied type of dynamic covalent bond, the oxime-carbamate bond. This type of bond is of interest, as it is characterised by chemistry that is different from that of classical carbamates and as it is sensitive to UV rays [105], which can lead to the formation of radicals that can be useful in the reworking phase. Also, the reaction intermediates proposed in this phase, such as isometric nitrone [106], promote a transcarbamylation different from that of the classical PU. Liu et al. [106], Lei et al. [105], and Zhang et al. [107] worked in this direction. The first one produced a poly(oxime-urethane) (POU), starting from HDI and various dioximes and trioximes without a catalyst. They obtained an elastomer-like polymer that can be reprocessed via hot-pressing at 120 °C and 10 MPa for 30 min, recovering up to 82% of the strain at break and 90% of the elongation at break compared to the pristine sample up to four times. Instead, the second group worked with a one-step reaction using polytetrahydrofuran ether diol, 3HDI, and diketone oxime at different molar ratios, obtaining a POU with excellent transparency. The fragmented network could be reprocessed via hot-pressing at 155 °C and 10 MPa for 40 min; and, for all the synthesised materials, an excellent recovery in tensile strength was achieved up to three times. Meanwhile, during the fourth cycle, a notable decrease was observed. The third group reacted polytetramethylene ether glycol, trimethylolpropane (TMP) as a crosslinker, IPDI, MDI, and dimethylglyoxime self-healing agent, in the presence of a bismuth acidic catalyst. The reprocessing was carried out on the material with a higher concentration of TMP; consequently, the most reticulated one, was hot-presses at 120 °C and 10 MPa for 3 min and subsequently heat-treated at 50 °C for 12 h to ensure the complete reaction of the oxime-carbamate bond. After three cycles, no variation in stress–strain curves and ATR-FTIR spectra were found, revealing the retention of the mechanical properties and structure of the pristine material, with complete re-formation of the oxime-carbamate bonds.

Liu et al. [108], instead, focused on a copolymer based on a combination of PHU and PTU, to produce poly(hydroxyurethane-co-mercaptotrithiourethane). Starting from the diglycidyl ether of bisphenol A, they made difunctional five-membered cyclic trithiocarbonate or carbonate, if reacting with CS_2_ or CO_2_, which were later combined by adding α,ω-diamino-terminated poly(propylene oxide), to obtain the copolymer chain. The final network was achieved by crosslinking the copolymers via a radical coupling approach, which generated disulphide bonds between the chains. The obtained material was hot-pressed at 90 °C for 30 min and reprocessed to obtain monolithic bodies without discernible defects. The specimens could be reprocessed three times, obtaining overlapping stress–strain curves with the pristine one. Despite different bond types, the authors proposed reprocessing properties accounted for the dynamic exchange of disulphide bonds, which caused the reshuffle or rearrangement in the crosslinked networks.

## 9. Conclusions

Globally, plastic production continues to grow exponentially to meet the demand for versatile and low-cost materials, which leads to the consideration of more effective, convenient, and easily implemented recycling processes. Polyurethanes cover an important percentage of all the produced polymers; and, because of the wide choice of components used in synthesis, they can be used in many areas of daily life. Because of their inherent nature, they cannot, to date, be recovered like other bulk polymers, such as polyethylene, polypropylene, or other thermoplastic polymers. Still, thanks to the discovery of CANs, new frontiers have opened for rapid mechanical recycling. This type of network allows for a quick and efficient reworking of thermoset polymers, like thermoplastics, ensuring a final product similar to the starting one by implementing true recycling or even upcycling.

Starting from classically synthesised PUs, it was possible to observe how the urethane bonds in the lattice, under certain temperature and pressure conditions, can provide exchange reactions, ensuring the macroscopic transformation of the reworked specimens. For example, for classical thermosets, elastomers, and PUs containing bio-derivative precursors, it was possible to implement reprocessing based primarily on hot-pressing techniques. Retention of the crosslink density and mechanical and thermal properties were also observed for up to five reworking cycles. The introduction of other molecules capable of forming different types of covalent bonds (e.g., disulphide bonds) in the network proved successful for obtaining reworkable lattices. The development of a network with an excess of hydroxyl groups (PHU case) successfully led to materials that were reworkable without the support of a catalyst. Following these hints, lattices based on other types of bonds that proved to be as dynamic as the urethane one have been developed. PTU exploited the presence of sulphur instead of oxygen in the repetitive unit to lower lattice activation energies and improve reworking conditions. Its lability can also be used to obtain reprocessed materials with superior properties compared to those of pristine materials. The insertion of another bond, such as the urea bond, in PUUs, further improved the materials by introducing new dynamic interactions, allowing reworking under harsher conditions. Reinforced PUs or composites with properties different from those of classic PUs have also been explored, obtaining interesting materials but highlighting critical issues in reworking owing to the insertion of certain fillers (e.g., cellulose and lignin).

The goal of identifying a common thread among the employed components was not realised. Finding relationships between both components proved tough. It was easy to see how, while starting with the same chemicals, the post-processing output varied in some circumstances. As a result, the acquisition of information on a priori reworking ability by analysing the proposed reagents does not appear to be achievable. Other parameters must be used to obtain information about potential reworkability and related reprocessing conditions. As hypothesised by Lagron et al. [109], these could be the mechanical properties of the materials themselves. According to the authors, rheological measures would allow the analysis of relaxation times and activation energies. This combined information would provide information for determining whether the material under evaluation was reworkable. Building on these findings for future research, the establishment of links between the reagents utilised and the ultimate qualities of the material may be possible. This could be a method for determining the likelihood of recycling a material prior to its synthesis.

Most of the work presented in the Review bases reprocessing on extremely simple techniques: reduction of the sample into small pieces or powders and subsequent hot-pressing or moulding; meanwhile, few are based on extrusion processes. In fact, to apply alternative reworking processes, one must be clear about the material’s behaviour, such as the change in viscosity after heating. For extrusion procedures, for example, this parameter is critical, and information about it might be linked to mechanical property evaluation. In this context, more research should be undertaken to determine which parameters truly affect the dynamicity of the system to identify thresholds for the application of various mechanical recycling procedures. This should be considered for future studies, as implementing new mechanisms or techniques could be the key to further lowering costs and making this recycling appealing at the industrial level. Based on the processes already in use, this would result in shorter recycling periods and lower temperatures. In truth, the simplicity of reprocessing cannot be attributed exclusively to reduced reprocessing temperatures. Lowering these significantly would result in the development of materials that are less expensive to reprocess but may not have any application market range. Too low reprocessing temperatures would imply dynamism at the material’s expected use temperatures, damaging its mechanical characteristics and, thus, rendering it not applicable. Therefore, studies on the real reasons for the ease of exchange between crosslinks need to be conducted.

Also, regarding the materials used in the studies reported in this Review, few use post-consumer or post-industrial PUs. Mostly, potentially more efficient lattice materials are synthesised from scratch, and their reworking is tested. Future studies should focus more on existing PUs or consider and evaluate the effect of other components in the formulation (e.g., fillers, stabilisers, and UV protectants) to consider potential applications outside the laboratory scale. Furthermore, a suitable approach that could be applied to this kind of materials, as reported in certain foam research, would be post-synthetic catalyst introduction. This would go a long way towards promoting lattice dynamism. This approach, however, may be unappealing because of the high cost and extensive usage of solvents required to swell the material. One approach to reworking materials that are not naturally dynamic could be the development of composites. It would be feasible to introduce dynamic bonds or strengthen those that currently exist with the addition of other components. Furthermore, the insertion of specific components may allow for reworking using less energy-intensive processes, like UV irradiation, as reported elsewhere in the paper, ultrasound, and microwaves [110].

Nonetheless, PU-CANs represent an extremely interesting and topical category of polymers. This study may be crucial for developing recyclable thermoset polymers with a view to sustainable development focused on recycling and reusing existing materials.

## Data Availability

Not applicable.

## Abbreviations

3HDI	Hexamethylene diisocyanate trimer
BDB	2,2′-(1,4-phenylene)-bis[4-mercaptan-1,3,2-dioxaborolane]
BDO	1,4-Butanediol
BHMF	2,5-bis-(hydroxymethyl)furan
CAN	Covalent Adaptable Network
CASE	Coatings, adhesives, sealants, and elastomers
CSBO	Carbonated soybean oil
CTO	Castor oil
D-A	Dies-Alder
DABCO	1,4-diazabicyclo [2.2.2] octane
DBDA	N,N’-di-tert-butylethylenediamine
DBTDL	Dibutyltin dilaurate
DCM	Dichloromethane
DDSQ	Double-decker silsesquioxane
DMA	Dynamic Mechanical Analysis
FTIR	Fourier Transformed Infrared Spectroscopy
GLY	Glycerine
HDI	Hexamethylene diisocyanate
HEDS	2-hydroxyethyl disulphide
HPS	Bis(4-hydroxyphenyl) disulphide
HTBD	Hydroxy-terminated polybutadiene
IPDI	Isophorone diisocyanate
IR	Infrared
MDI	Methylene diphenyl diisocyanate
NCC	nanocrystalline cellulose
NIR	Near Infrared
NPs	Nano particles
PCL	Poly(ε-caprolactone)
PEG	Polyethylene glycol
PHU	Polyhydroxyurethanes
POU	Poly(oxime-urethane)
PPG	Poly(propylene glycol)
PTMEG	Polytetramethylene ether glycol
PTU	Polythiourethanes
PU	Polyurethanes
PUF	Polyurethanes foam
PUU	Poly(urethane-urea)
PVPy	N-vinylpyrrolidone
ROMP	Ring-opening metathesis polymerisation
SEC	Sorbitol ether carbonate
TDI	Toluene diisocyanate
TEG	Tetraethylene glycol
TMMP	Trimethylolpropane tris(3-mercaptopropionate)
TMP	Trimethylolpropane
UPy	Ureidopyrimidinone
UV	Ultraviolet
